# Plasmacytoid dendritic cells orchestrate TLR7-mediated innate and adaptive immunity for the initiation of autoimmune inflammation

**DOI:** 10.1038/srep24477

**Published:** 2016-04-14

**Authors:** Hideaki Takagi, Keiichi Arimura, Tomofumi Uto, Tomohiro Fukaya, Takeshi Nakamura, Narantsog Choijookhuu, Yoshitaka Hishikawa, Katsuaki Sato

**Affiliations:** 1 Division of Immunology, Department of Infectious Diseases, Faculty of Medicine, University of Miyazaki, 5200 Kihara, Kiyotake, SY 889-1692, Japan; 2 Department of Oral and Maxillofacial Surgery, Faculty of Medicine, University of Miyazaki, 5200 Kihara, Kiyotake, Miyazaki 889-1692, Japan; 3 Department of Otolaryngology, Head and Neck Surgery, Faculty of Medicine, University of Miyazaki, 5200 Kihara, Kiyotake, Miyazaki 889-1692, Japan; 4 Division of Histochemistry and Cell Biology, Department of Anatomy, Faculty of Medicine, University of Miyazaki, Miyazaki 889-1692, Japan; 5 Japan Science and Technology Agency, Precursory Research for Embryonic Science and Technology (PRESTO), 4-1-8 Hon-cho, Kawaguchi, Saitama 332-0012, Japan

## Abstract

Endosomal toll-like receptor (TLR)-mediated detection of viral nucleic acids (NAs) and production of type I interferon (IFN-I) are key elements of antiviral defense, while inappropriate recognition of self NAs with the induction of IFN-I responses is linked to autoimmunity such as psoriasis and systemic lupus erythematosus. Plasmacytoid dendritic cells (pDCs) are cells specialized in robust IFN-I secretion by the engagement of endosomal TLRs, and predominantly express sialic acid-binding Ig-like lectin (Siglec)-H. However, how pDCs control endosomal TLR-mediated immune responses that cause autoimmunity remains unclear. Here we show a critical role of pDCs in TLR7-mediated autoimmunity using gene-modified mice with impaired expression of Siglec-H and selective ablation of pDCs. pDCs were shown to be indispensable for the induction of systemic inflammation and effector T-cell responses triggered by TLR7 ligand. pDCs aggravated psoriasiform dermatitis mediated through the hyperproliferation of keratinocytes and enhanced dermal infiltration of granulocytes and γδ T cells. Furthermore, pDCs promoted the production of anti-self NA antibodies and glomerulonephritis in lupus-like disease by activating inflammatory monocytes. On the other hand, Siglec-H regulated the TLR7-mediated activation of pDCs. Thus, our findings reveal that pDCs provide an essential link between TLR7-mediated innate and adaptive immunity for the initiation of IFN-I-associated autoimmune inflammation.

Dendritic cells (DCs) known as essential antigen (Ag)-presenting cells (APCs) of the immune system efficiently recognize pathogens through pattern recognition receptors (PRRs) including Toll-like receptors (TLRs), secrete multiple cytokines and activate naïve T cells during primary responses^1^,^2^,^3^. The latter property distinguishes them from other innate immune cell types, and establishes a key link between innate and adaptive immunity^4^,^5^,^6^. DCs are represented by two major lineages, classical or conventional DCs (cDCs) and plasmacytoid DCs (pDCs)^1^,^2^,^3^. pDCs are specialized in endosomal TLR7/9-mediated recognition of viral nucleic acids (NAs) and respond with the massive secretion of type I IFN (IFN-I). Therefore, pDCs have been considered as important mediators of antiviral responses^7^,^8^,^9^.

While pDCs are identified by the combination of multiple cell surface molecules such as Gr-1 or bone marrow stromal antigen 2 (BST-2)^8^, sialic acid-binding immunoglobulin (Ig)-like lectin-H (Siglec-H), which is unique among Siglec proteins in that it associates with the adaptor protein DNAX-activation protein 12 (DAP12), is predominantly found on the cell surface of pDCs in lymphoid organs^10^,^11^,^12^. For precise evaluation of the contribution of pDCs to the immune system, we have recently engineered knock-in (KI) mice that express the diphtheria toxin (DT) receptor (DTR) under the control of the *Siglech* gene, in which the DTR-containing KI cassette was introduced into the 3′ untranslated region (UTR) of the Siglech gene to produce *Siglech*^dtr/dtr^ KI mice^13^. *Siglech*^dtr/dtr^ KI mice harbor the impaired expression of Siglec-H on pDCs due to disruption of the *Siglech* open reading frame, leading to knock-down (kd) of its transcriptional expression (referred to as *Siglech*^kd^ mice), whereas pDCs in *Siglech*^dtr/+^ KI mice retain its expression. More importantly, *Siglech*^dtr/dtr^ KI mice exhibit >97% depletion of pDCs in lymphoid tissues within 24 hrs after initial DT treatment and tolerate repeated treatments with no harmful observation, allowing long-term depletion of pDCs (referred to as pDC-ablated mice). Using these KI mice, we have demonstrated that pDCs play a crucial role in the control of inflammation and T-cell responses triggered by microbial injection *in vivo*, and Siglec-H controls their function.

It has become apparent that erroneous recognition of self NAs by endosomal TLRs is involved in IFN-I-associated autoimmune diseases such as psoriasis^14^,^15^,^16^,^17^,^18^ and systemic lupus erythematosus (SLE)^14^,^15^,^19^,^20^,^21^, in which the role of TLR7 predominates over that of TLR9 in the pathogenesis^22^,^23^. Recent studies have suggested that the activation of pDCs by recognition of self NA causes elevated IFN-I levels, and this process contributes to the induction of pathogenesis in psoriasis^24^ and SLE^25^,^26^,^27^. Although pDCs have been proposed as a key source of aberrant IFN-I production in psoriasis and SLE, how they control innate and adaptive immune responses leading to the initiation of IFN-I-associated autoimmune diseases is poorly understood.

In this study, we show that pDCs are indispensable for the TLR7-mediated development of I-IFN-associated psoriasis-like skin inflammation and lupus-like systemic autoimmune disease under the intrinsic Siglec-H-mediated control of inflammation and T-cell responses.

## Results

### Siglec-H suppresses TLR7-mediated activation of pDCs

**Figure 1:**
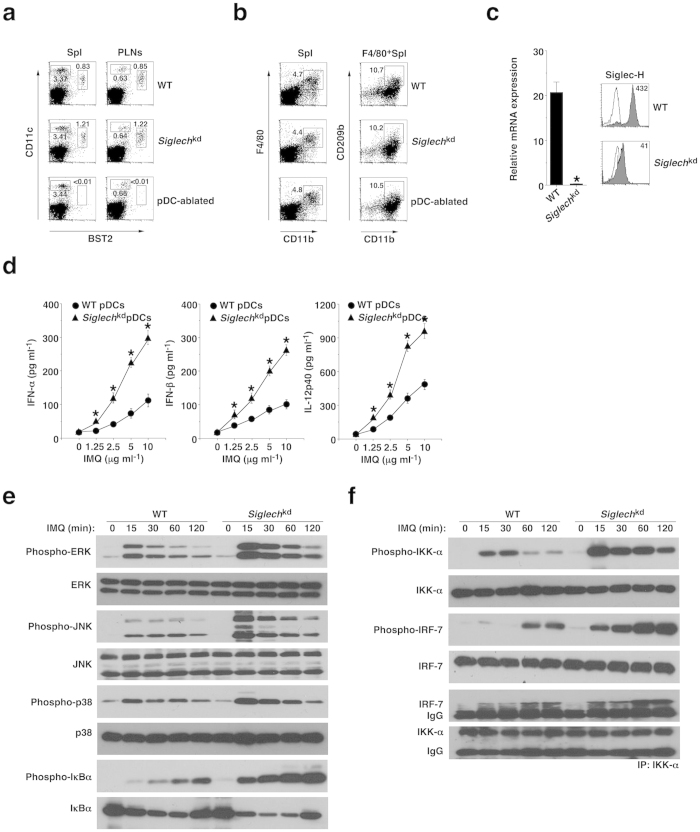
Deficiency of Siglec-H and ablation of pDCs in *Siglech*^dtr/dtr^ mice. (**a**) The frequency of CD11c^+^BST2^+^ pDCs in Spl and PLNs obtained from the C57BL/6-background WT mice (n = 3), *Siglech*^kd^ mice (n = 3), and pDC-ablated mice (n = 3) was analyzed by flow cytometry. Data are presented as a dot plot, and numbers represent the proportion of CD11c^+^BST2^+^ pDCs among leukocytes in each quadrant. (**b**) The frequency of CD11b^+^F4/80^+^CD209b^+^ macrophages in Spl obtained from WT mice (n = 3), *Siglech*^kd^ mice (n = 3) and pDC-ablated mice (n = 3) was analyzed by flow cytometry. Data are presented as a dot plot, and numbers represent the proportion of CD11b^+^F4/80^+^ macrophages among leukocytes (left panel) and CD11b^+^CD209b^+^ macrophages among F4/80^+^ macrophages (right panel) in each quadrant. (**c**) Transcriptional expression (left panel) or cell surface expression (right panel) of Siglec-H in pDCs obtained from WT mice (n = 3) and *Siglech*^kd^ mice (n = 3) was measured by quantitative reverse transcriptase (RT)-PCR or flow cytometry. (Left panel) Data are the mean ± s.d. from three individual samples in a single experiment. (Right panel) Data are presented by a histogram, and numbers represent mean fluorescence intensity (MFI). (**d**) pDCs obtained from WT mice (n = 3) and *Siglech*^kd^ mice (n = 3) were stimulated or not stimulated with IMQ, and the production of cytokines was measured by enzyme-linked immunosorbent assay (ELISA). Data are the mean ± s.d. from three individual samples in a single experiment. *P < 0.01 compared with WT mice. (**e**,**f**) pDCs obtained from WT mice (n = 3) and *Siglech*^kd^ mice (n = 3) were stimulated or not stimulated with IMQ for the period indicated, at which time cells were lysed. Total lysate or the immunoprecipitate with anti-IKK-α Ab prepared from the pooled pDCs (each n = 3) was analyzed using Ab specific for ERK (**e**), JNK (**e**), p38 (**e**), IkBα (**e**), IKK-α (**f**) and IRF-7 (**f**), or for phosphorylated versions of these proteins. IP, immunoprecipitation. *P < 0.01 compared with WT mice. All data are representative of at least three independent experiments.

Consistent with our published report^13^, pDC-ablated mice (DT-treated *Siglech*^dtr/dtr^ KI mice) exhibited almost complete and specific elimination of CD11c^+^BST2^+^ pDCs among leukocytes in spleen (Spl) and peripheral lymph nodes (PLNs) under steady-state conditions, compared with wild-type (WT) mice (Fig. 1a and Supplementary Fig. 1a). Similar to the previous observation for almost normal histological localization of CD11b^+^F4/80^+^CD209b^+^ marginal zone macrophages (MZM) expressing intracellular Siglec-H^11^ in pDC-ablated mice^13^, flow cytometric analysis further confirmed that there was no substantial difference in the proportion and number of CD11b^+^F4/80^+^CD209b^+^ MZM between WT mice and pDC-ablated mice (Fig. 1b and Supplementary Fig. 1b). These observations are different from other inducible pDC-ablation models, in which both pDCs and MZM were depleted after DT administration^28^. As some DC progenitor (CDP) reportedly expressed Siglec-H in bone marrow (BM)^29^, pDC-ablated mice (DT-treated *Siglech*^dtr/+^ KI mice) exhibited the ablation of CDP expressing Siglec-H as well as pDCs, whereas they had the normal proportion of CD11c^high^BST2^−^ cDCs in Spl, PLNs, and BM (Supplementary Fig. 1c–f)^13^.

*Siglech*^kd^ pDCs displayed extremely low transcriptional and cell surface expression of Siglec-H compared with WT pDCs (Fig. 1c and Supplementary Fig. 1g). Furthermore, *Siglech*^kd^ pDCs showed lower expression of MHC II (I-A/I-E) than WT pDCs, although they had similar expression levels of costimulatory molecules under steady-state conditions (Supplementary Fig. 2a). On the other hand, the ability of *Siglech*^kd^ pDCs to activate OVA-specific OT-II CD4^+^ T cells^13^,^30^ was lower than that of WT pDCs, whereas there was no difference in the ability to activate OVA-specific OT-I CD8^+^ T cells^17^,^33^ between WT pDCs and *Siglech*^kd^ pDCs (Supplementary Fig. 2b,c). However, cDCs obtained from WT mice and *Siglech*^kd^ mice showed similar capacity to activate Ag-specific T cells (Supplementary Fig. 2d,e).

While WT pDCs produce IFN-α, IFN-β and interleukin (IL)-12p40 in response to imiquimod (IMQ), known as a synthetic TLR7 ligand^31^, *Siglech*^kd^ pDCs exhibited the further enhanced production of these cytokines after stimulation (Fig. 1d), indicating that Siglec-H suppresses the ability of pDCs to produce cytokines in response to TLR7 ligand. On the other hand, *Siglech*^kd^ pDCs not only showed higher phosphorylation of ERK, JNK and p38 but also exhibited greater phosphorylation and degradation of inhibitor of nuclear factor κB (NF-κB) (IκB), which indicate activation of NF-κB, than WT pDCs upon stimulation with IMQ (Fig. 1e). Furthermore, *Siglech*^kd^ pDCs displayed greater phosphorylation of IκB kinase (IKK)-α and the association of IKK-α with IFN regulatory factor (IRF)-7 following stimulation with IMQ than WT pDCs (Fig. 1f). These results indicate that Siglec-H suppresses the TLR7-mediated activation of the signaling cascades for NF-κB and IRF-7, which are reportedly critical for the production of proinflammatory cytokines and IFN-I in pDCs^32^.

### pDCs exacerbates TLR7-mediated systemic inflammatory responses *in vivo*

**Figure 2:**
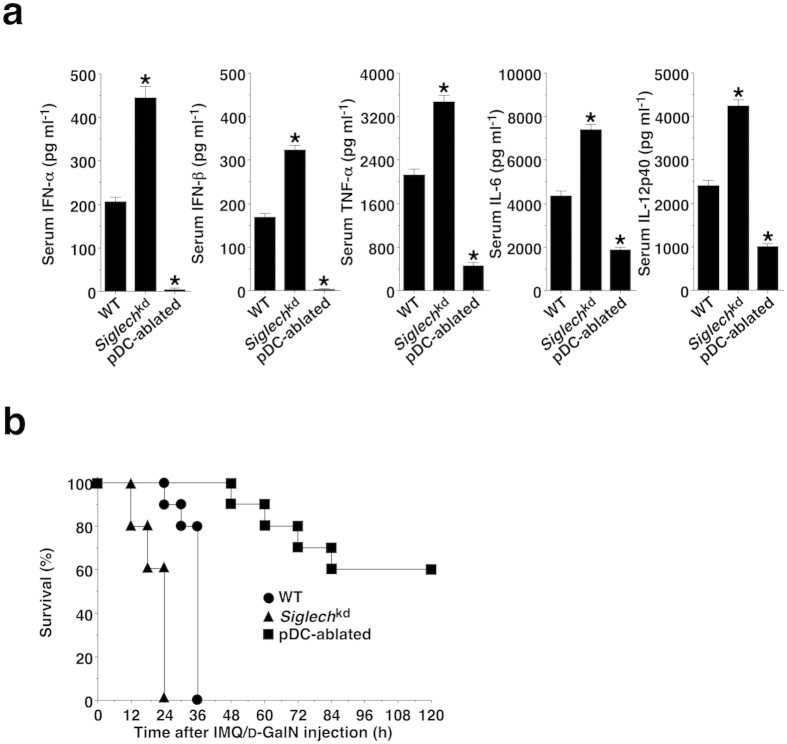
pDCs induce TLR7-mediated systemic lethal inflammation. The C57BL/6-background WT mice (n = 10), *Siglech*^kd^ mice (n = 10), and pDC-ablated mice (n = 10) were injected with IMQ plus D-GalN. (**a**) Serum production of cytokines was measured 24 hrs after injection with IMQ plus D-GalN by ELISA. Data are the mean ± s.d. from ten individual samples in a single experiment. (**b**) Survival rate was monitored at the indicated times for 120 hrs after the injection of IMQ plus D-GalN. *P < 0.01 compared with WT mice. All data are representative of at least three independent experiments.

We addressed the roles of pDCs in the TLR7-mediated inflammatory response *in vivo*. After the administration of IMQ and D-galactosamine (D-GalN), *Siglech*^kd^ mice displayed increased levels of IFN-α, IFN-β, TNF-α, βIL-6 and IL-12p40, while pDC-ablated mice exhibited a dramatic reduction of serum cytokine production compared with WT mice (Fig. 2a). Furthermore, *Siglech*^kd^ mice or pDC-ablated mice were more susceptible or resistant to the TLR7-mediated lethality than WT mice (Fig. 2b). However, similar serum cytokine production and the lethality were observed among these groups of mice that had been treated with LPS and D-GalN (Supplementary Fig. 3). These results indicate that pDCs are required for initiation of TLR7-mediated systemic inflammatory response *in vivo*.

**Figure 3:**
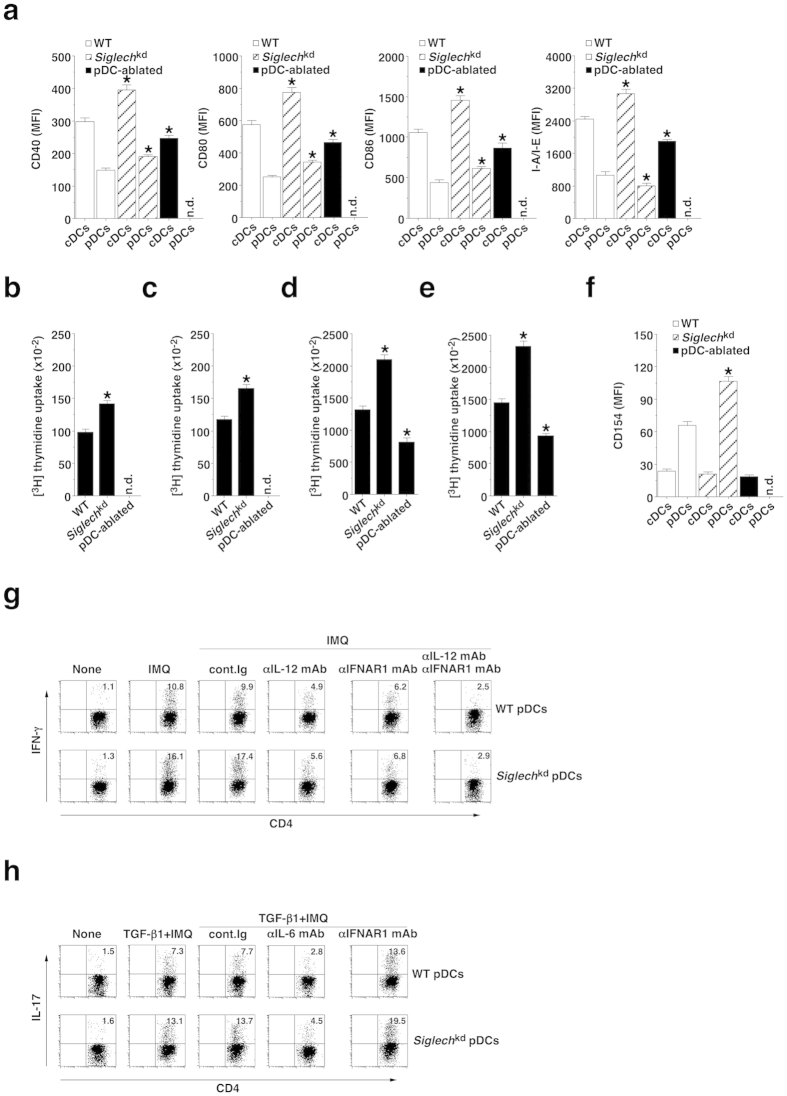
Siglec-H regulates TLR7-mediated activation of pDCs. (**a–f**) The C57BL/6-background WT mice (n = 6), *Siglech*^kd^ mice (n = 6), and pDC-ablated mice (n = 6) were injected with or without IMQ, and cDCs and pDCs were obtained 24 hrs after injection. (**a**) The expression of MHC and costimulatory molecules on cDCs and pDCs was analyzed by flow cytometry. Data are MFI ± s.d. from six individual samples in a single experiment. (**b–e**) CD45.1 OT-II CD4^+^ T cells (**b**,**d**) or CD45.1 OT-I CD8^+^ T cells (**c**,**e**) were cultured with pDCs (**b,c**) or cDCs (**d**,**e**) in the presence or absence of OVA_323–339_ peptide (**b**,**d**) or OVA_257–264_ peptide (**c**,**e)**, and the proliferation was measured by [^3^H]thymidine incorporation. Data are the mean ± s.d. from six individual samples in a single experiment. (**f**) The expression of CD154 on cDCs and pDCs was analyzed by flow cytometry. Data are MFI ± s.d. from six individual samples in a single experiment. (**g**,**h)** CD45.1^+^ OT-II CD4^+^ T cells were cultured with pDCs obtained from WT mice (n = 3) and *Siglech*^kd^ mice (n = 3) in the presence or absence of IMQ in combination with OVA_323-339_ peptide and various blocking mAbs under T_H_1 (**g**)- or T_H_17 (**h**)-polarized culture conditions for 3 days, and intracellular production of IFN-γ (**g**) or IL-17 (**h**) in the cultured CD4^+^ T cells was analyzed by flow cytometry. Data are presented as a dot plot, and numbers represent the proportion of IFN-γ^+^ cells (**g**) and IL-17^+^ cells (**h**) among gated CD4^+^ T cells in each quadrant. *P < 0.01 compared with WT mice. All data are representative of at least three independent experiments.

Whereas the administration of IMQ increased the expression of MHC II and costimulatory molecules on cDCs and pDCs, and their ability to activate OT-II CD4^+^ T cells and OT-I CD8^+^ T cells in WT mice, their expression levels of these molecules and T-cell activation were further enhanced in *Siglech*^kd^ mice (Fig. 3a–e). In contrast, cDCs obtained from pDC-ablated mice showed lower expression of these molecules and ability to activate OT-II CD4^+^ T cells and OT-I CD8^+^ T cells than those obtained from WT mice (Fig. 3a–e). However, there was no significant difference in the proprotion of cDCs in Spl and PLNs (approximately 3.4% and 0.6%, respectively) among these groups of mice. We also observed that the TLR7-mediated upregulation of the expression of MHC II and costimulatory molecules on cDCs and pDCs as well as their ability to activate Ag-specific T cells was reduced in *Ifnar1*^−/−^ mice^33^ and *Cd40*^−/−^ mice^34^ when compared with WT mice (Supplementary Fig. 4). Although both cDCs and pDCs did not express CD154 under steady-state conditions, the administration of IMQ also induced the expression of CD154 (CD40 ligand) on pDCs, but not on cDCs, in WT mice, and the expression level of CD154 on pDCs was higher in *Siglech*^kd^ mice than in WT mice (Fig. 3f). These results indicate that pDCs promote the activation status of cDCs under TLR7-mediated inflammatory conditions.

*In vitro* analysis revealed that *Siglech*^kd^ pDCs displayed an enhanced capacity to generate IFN-γ-producing CD4^+^ T cells (T helper 1; T_H_1 cells)^35^ and IL-17-producing CD4^+^ T cells (T_H_17 cells)^35^ compared with WT pDCs (Fig. 3g,h and Supplementary Fig. 5a,b), indicating that Siglec-H suppresses the capacity of pDCs to induce T_H_1 cells and T_H_17 cells. Furthermore, the differentiation of these T_H_1 cells was blocked by anti-IL-12 monoclonal Ab (mAb) and anti-IFN-α/β receptor I (IFNAR1) mAb, whereas the generation of these T_H_17 cells was suppressed or enhanced by anti-IL-6 mAb or anti-IFNAR1 mAb (Fig. 3g,h and Supplementary Fig. 5a,b). While cDCs had a more potent ability to generate T_H_1 cells and T_H_17 cells than pDCs in WT mice, similar effects of these mAbs on the generation of these CD4^+^ effector T (T_eff_) cells was observed (Supplementary Fig. 5c–f).

pDCs obtained from *Tlr7*^−/−^ mice^31^, *Ifnar1*^−/−^ mice and *Cd40*^−/−^ mice showed reduced generation of T_H_1 cells, whereas pDCs obtained from *Tlr7*^−/−^ mice and *Cd40*^−/−^ mice or *Ifnar1*^−/−^ mice exhibited reduced or enhanced differentiation of T_H_17 cells when compared with those obtained from WT mice (Supplementary Fig. 6). These results indicate that pDCs generate T_H_1 cells and T_H_17 cells, possibly mediated through the cytokine production and costimulation.

### pDCs participated in the induction of T-cell responses *in vivo*

**Figure 4:**
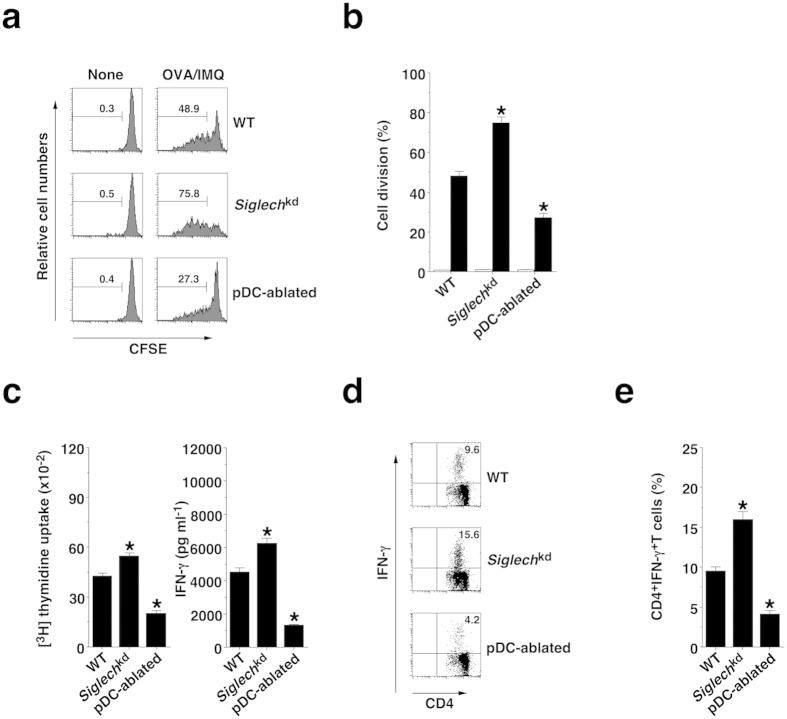
pDCs promote TLR7-mediated Ag-specific CD4^+^ T-cell responses *in vivo*. (**a**,**b**) CFSE-labeled CD45.1^+^OT-II CD4^+^ T cells were transferred into the C57BL/6-background WT mice (n = 6), *Siglech*^kd^ mice (n = 6), and pDC-ablated mice (n = 6), and then the mice were immunized with IMQ plus OVA protein. Ag-specific division of CD45.1^+^OT-II CD4^+^ T cells was analyzed 3 days after the immunization by flow cytometry. (**a**) Data are presented as a histogram, and numbers represent the proportion of the dividing cells among gated CD45.1^+^OT-II CD4^+^ T cells in each histogram. (**b**) Data are the mean percentage of positive cells ± s.d. from six individual samples in a single experiment. (**c**–**e**) The C57BL/6-background WT mice (n = 6), *Siglech*^kd^ mice (n = 6), and pDC-ablated mice (n = 6) were immunized with OVA protein plus IMQ. At 14 days after the immunization, Spl CD4^+^ T cells were cultured with WT CD11c^+^ DCs in the presence or absence of OVA protein for the measurement of proliferative responses by [^3^H]thymidine incorporation ((**c**) left panel) and production of IFN-γ ((**c**) right panel) by ELISA. (**d**,**e**) Intracellular production of IFN-γ in the cultured CD4^+^ T cells was analyzed by flow cytometry. (**d**) Data are presented as a dot plot, and numbers represent the proportion of IFN-γ^+^ cells among gated CD4^+^ T cells in each quadrant. (**e**) Data are the mean percentage of positive cells ± s.d. from six individual samples in a single experiment. *P < 0.01 compared with WT mice. All data are representative of at least three independent experiments.

To address the roles of pDCs in the induction of Ag-specific proliferation of CD4^+^ T cells *in vivo*, carboxyfluorescein diacetate-succinimidyl ester (CFSE)-labeled OT-II CD4^+^ T cells were adoptively transferred into mice, and their division was analyzed 3 days after the systemic injection of soluble OVA protein plus IMQ (Fig. 4a,b). When compared with Ag-specific division of CD4^+^ T cells in WT mice, *Siglech*^kd^ mice or pDC-ablated mice exhibited enhancement or reduction of this response.

After immunization with OVA protein plus IMQ, CD4^+^ T cells from *Siglech*^kd^ mice or pDC-ablated mice showed higher or lower proliferation and production of IFN-γ as well as frequency of T_H_1 cells than CD4^+^ T cells from WT mice (Fig. 4c–e). On the other hand, *Tlr7*^−/−^ mice, *Ifnar1*^−/−^ mice and *Cd40*^−/−^ mice showed reduced activation of Ag-specific CD4^+^ T cells compared with WT mice (Supplementary Fig. 7).

**Figure 5:**
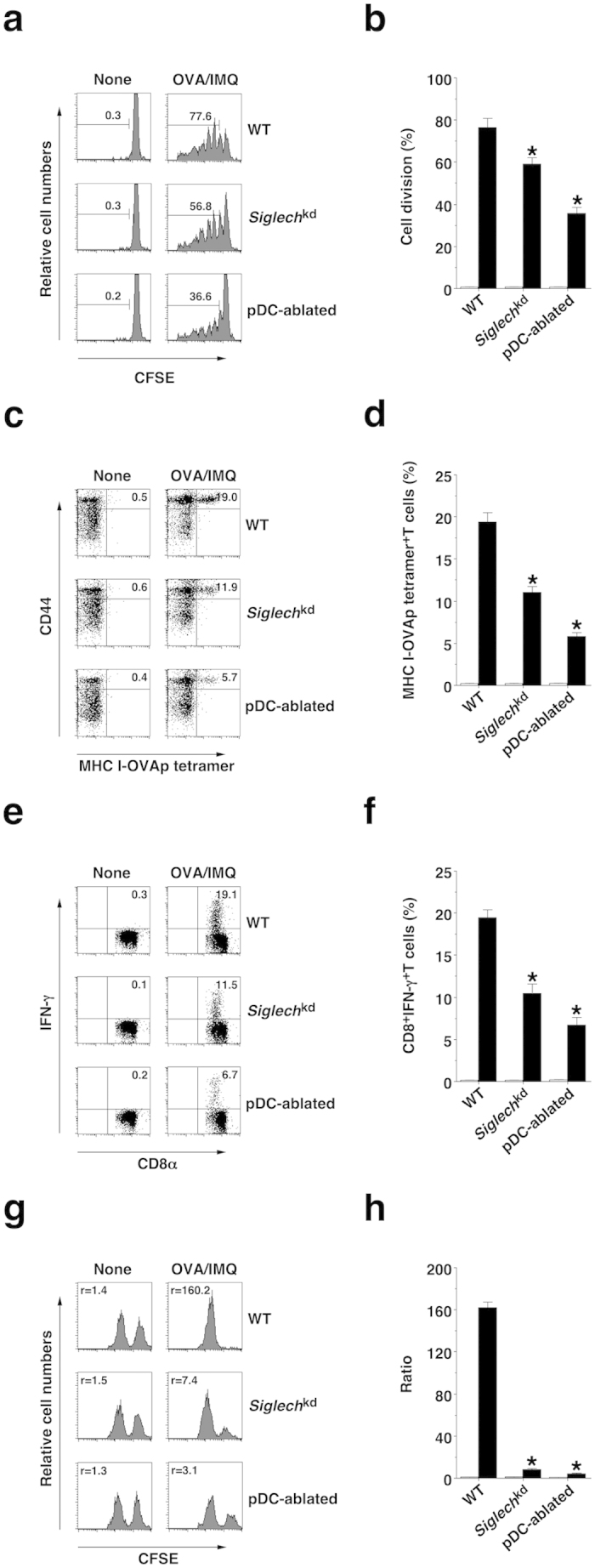
pDCs promote TLR7-mediated Ag-specific CD8^+^ T-cell responses *in vivo*. (**a**,**b**) CFSE-labeled CD45.1^+^OT-I CD8^+^ T cells were transferred into the C57BL/6-background WT mice (n = 6), *Siglech*^kd^ mice (n = 6), and pDC-ablated mice (n = 6), and then the mice were immunized with OVA protein plus IMQ. Ag-specific division of CD45.1^+^OT-I CD8^+^ T cells was analyzed 3 days after the immunization by flow cytometry. (**a**) Data are presented as a histogram, and numbers represent the proportion of the dividing cells among gated CD45.1^+^OT-I CD8^+^ T cells in each histogram. (**b**) Data are the mean percentage of positive cells ± s.d. from six individual samples in a single experiment. (**c–h**) The C57BL/6-background WT mice (n = 6), *Siglech*^kd^ mice (n = 6), and pDC-ablated mice (n = 6) were immunized with OVA protein, IMQ and anti-CD40 mAb, and then a mixture of unpulsed CFSE^low^ cells plus Ag-pulsed CFSE^high^ cells was injected 5 days after the immunization. At 6 days after the immunization, splenocytes were analyzed for the generation of MHC I-OVA tetramer^+^CD44^high^CD8^+^ T cells (**c,d**), for intracellular IFN-γ-producing CD8^+^ T cells (**e**,**f**) and for cytotoxic activity *in vivo* (**g**,**h**) by flow cytometry. Data are presented as a dot plot (**c**,**e**), and numbers represent the proportion of MHC I-OVA tetramer^+^CD44^high^ cells (**c**) and IFN-γ^+^ cells (**e**) among gated CD8^+^ T cells in each quadrant, or by a histogram (**g**), and numbers represent the ratio of unpulsed CFSE^low^ cells to Ag-pulsed CFSE^high^ cells in each histogram. (**d**,**f**,**h**) Data are the mean percentage of positive cells (**d**,**f**) or ratio (**h**) ± s.d. from six individual samples in a single experiment. *P < 0.01 compared with WT mice. All data are representative of at least three independent experiments.

Upon immunization with OVA protein plus IMQ, *Siglech*^kd^ mice and pDC-ablated mice displayed reduction of Ag-specific division of CD8^+^ T cells through cross-presentation pathway^6^ compared with WT mice (Fig. 5a,b).

Similarly, WT mice showed efficient generation of MHC I-OVA tetramer^+^CD44^high^CD8^+^ T cells and CD8^+^IFN-γ^+^ T cells as well as their significant cytotoxic activity against targeted cells after immunization with OVA protein combined with IMQ and anti-CD40 mAb, whereas *Siglech*^kd^ mice and pDC-ablated mice had a reduction in the generation of OVA-specific CTLs (Fig. 5c–h). On the other hand, *Tlr7*^−/−^ mice, *Ifnar1*^−/−^ mice and *Cd40*^−/−^ mice showed impaired generation of OVA-specific CTLs compared with WT mice (Supplementary Fig. 8).

Collectively, these results indicate that pDCs potentiate Ag-specific T-cell responses under IMQ-induced inflammatory conditions *in vivo*.

### pDCs exacerbate IMQ-induced psoriasiform dermatitis

**Figure 6:**
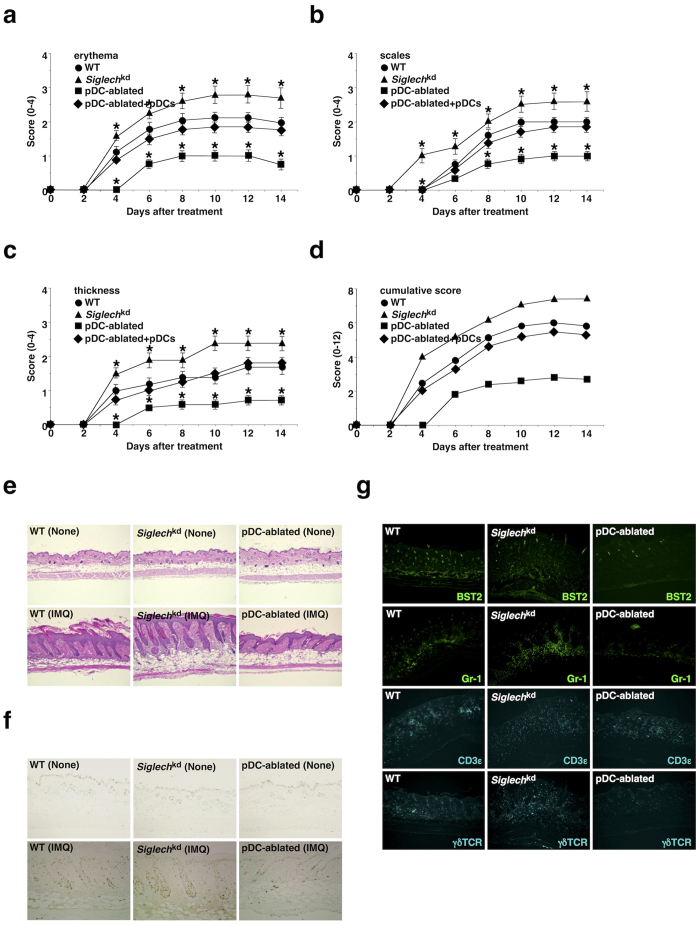
pDCs aggravate IMQ-induced psoriasiform dermatitis. The C57BL/6-background WT mice (n = 10), *Siglech*^kd^ mice (n = 10), and pDC-ablated mice (n = 10) that had been adoptively transferred with or without pDCs were treated topically with IMQ cream on the shaved back every other day for 14 days. (**a–d**) The disease severity of each mouse was scored daily, and erythema (**a**), scaling (**b**), thickness (**c**) and cumulative score (**d**) of the back skin at the indicated times were plotted. Data are the mean ± s.d. from ten individual samples in a single experiment. *P < 0.01 compared with WT mice. (**e**) H&E staining of the paraffin-embedded sections (magnification; 10×) obtained from the back skin of untreated mice (upper panel) and mice 6 days after treatment with IMQ cream (lower panel). (**f**) Immunohistochemical detection of proliferating cell nuclear Ag (PCNA) was performed on paraffin-embedded sections (magnification; 10×) obtained from the back skin of untreated mice (upper panel) and mice 6 days after treatment with IMQ cream (lower panel). (**g**) Immunofluorescent microscopic analysis was performed on frozen horizontal sections (magnification; 10×) obtained from the back skin of mice 6 days after treatment with IMQ cream. Sections were stained for BST2 (green), Gr-1 (green), CD3ε (cyan) or γδTCR (cyan). All data are representative of at least three independent experiments.

Although psoriasiform dermatitis is reportedly associated with the increased secretion of IFN-I and several proinflammatory cytokines as well as the massive infiltration of inflammatory leukocytes in addition to the hyperproliferation and disturbed differentiation of keratinocytes (parakeratosis), leading to thickening of the epidermis (acanthosis)^14^,^15^,^16^,^17^,^18^, how immune responses caused the psoriatic inflammation of skin is poorly understood. We therefore examined the pathological mechanisms of psoriasis using a murine model of IMQ-induced psoriasiform dermatitis^36^,^37^,^38^. Repetitive topical application of IMQ on the shaved back skin of WT mice led to the development of psoriasiform inflammation with significant erythema, scaling and thickening (Fig. 6a–d and Supplementary Fig. 9). Analysis of sections of skin obtained from IMQ-treated WT mice showed a phenomenon typical of psoriasis skin lesions, in which increased epidermal thickening in skin was caused by hyperproliferation of keratinocytes in the basal cell layer, and the scaling of the skin as an indication of parakeratosis was associated with the altered epidermal differentiation (Fig. 6e,f). Immunohistochemical analysis also demonstrated that leukocytes, including BST2^+^ pDCs, Gr-1^+^ granulocytes and γδTCR^+^ T cells, infiltrated into the skin of WT mice (Fig. 6g).

We examined the roles of TLR7, IFN-I, and costimulatory molecules in the development of psoriasiform skin inflammation. IMQ-treated *Tlr7*^−/−^ mice exhibited impaired progression of psoriatic inflammation compared with IMQ-treated WT mice (Supplementary Fig. 10), indicating that the development of IMQ-induced skin inflammation is largely dependent on TLR7-mediated signaling. While the disease severity observed in IMQ-treated *Ifnar1*^−/−^ mice was less than that in IMQ-treated WT mice until 6 days after the initial application of IMQ, they exhibited comparable disease onset at 14 days (Supplementary Fig. 10). These results indicate that the activation of IFN-I signaling contributes to the primary events in the cascade initiating psoriatic inflammation, whereas it is dispensable for the development of psoriatic lesions. On the other hands, *Cd40*^-/-^ mice showed greater reduction in the development of psoriasiform skin inflammation than *Cd80*^−/−^*Cd86*^−/−^ mice^35^ when compared with WT mice (Supplementary Fig. 11), indicating that costimulation between T cells and APCs is necessary for the formation of psoriasiform dermatitis.

We also examined the requirement for CD4^+^ T_eff_ cells in the development of psoriatic disease (Supplementary Fig. 12). IMQ-treated *Rag2*^−/−^γ*c*^−/−^ mice, which are completely devoid of T cells, B cells and NK cells, displayed marked reduction in the severity of skin inflammation compared with IMQ-treated WT mice, and reconstitution with naïve CD4^+^ T cells restored this response, indicating that CD4^+^ T cells are required for disease progression. Furthermore, IMQ-treated *Rag2*^−/−^γ*c*^−/−^ mice that had been adoptively transferred with T_H_17 cells showed greater disease severity than those with T_H_1 cells when compared with IMQ-treated WT mice. These results indicate that T_H_17 cells rather than T_H_1 cells are crucial for the development of psoriatic inflammation.

We further assessed the role of pDCs in the initiation and progression of IMQ-induced psoriasiform dermatitis. *Siglech*^kd^ mice or pDC-ablated mice showed accelerated or attenuated development of IMQ-induced psoriasiform inflammation, while the reconstitution of pDC-ablated mice with WT pDCs restored this pathogenesis (Fig. 6a–d and Supplementary Fig. 9). Furthermore, IMQ-treated *Siglech*^kd^ mice displayed more significant acanthosis and parakeratosis as well as infiltration of mononuclear cells into the skin, whereas IMQ-treated pDC-ablated mice exhibited less psoriasiform-associated skin pathogenesis than IMQ-treated WT mice (Fig. 6e–g). We observed that IMQ-treated cDC-ablated mice (CD11c-DTR mice)^39^ also exhibited lower severity of skin inflammation than IMQ-treated WT mice, and IMQ-treated cDC/pDC-ablated mice had more dramatic reduction in the progression of psoriatic inflammation (Supplementary Fig. 13). These results indicate that pDCs as well as cDCs are indispensable for the initiation and progression of the local skin inflammation in IMQ-induced psoriasis.

### pDCs promote IMQ-induced skin-associated inflammation and T-cell responses

**Figure 7:**
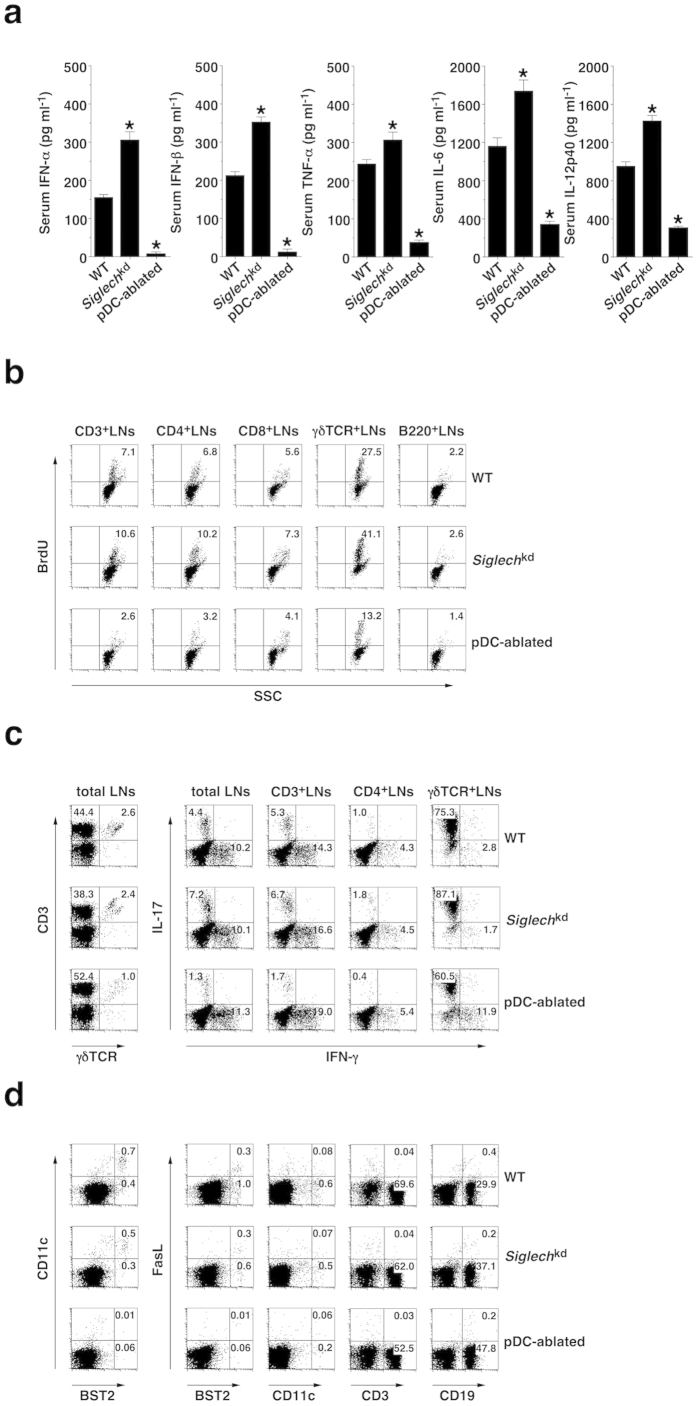
pDCs enhance IMQ-induced inflammation and T-cell responses. The C57BL/6-background WT mice (n = 6), *Siglech*^kd^ mice (n = 6), and pDC-ablated mice (n = 6) were treated topically with IMQ cream on the shaved back every other day for 6 days. (**a**) Serum production of cytokines was measured 6 days after treatment with IMQ cream by ELISA. Data are the mean ± s.d. from six individual samples in a single experiment. *P < 0.01 compared with WT mice. (**b**) The frequency of BrdU^+^ lymphocyes in skin-draining LNs was analyzed 6 days after treatment with IMQ cream by flow cytometry. Data are presented as a dot plot, and numbers represent the proportion of positive cells in each quadrant. (**c**) The constituency of T-cell subsets in skin-draining LNs (left panel) and their intracellular production of IFN-γ and IL-17 (right panel) were analyzed 6 days after treatment with IMQ cream by flow cytometry. Data are presented as a dot plot, and numbers represent the proportion of positive cells in each quadrant. (**d**) The expression of FasL and cell surface molecules on leukocytes in skin-draining LNs was analyzed 6 days after treatment with IMQ cream by flow cytometry. Data are presented as a dot plot, and numbers represent the proportion of positive cells in each quadrant. All data are representative of at least three independent experiments.

We examined how pDCs control immune responses leading to the initiation of psoriatic inflammation. Following the topical application of IMQ, *Siglech*^kd^ mice or pDC-ablated mice exhibited higher or lower serum production of IFN-α, IFN-β, TNF-α, βIL-6 and IL-12p40 than WT mice (Fig. 7a). Furthermore, *Siglech*^kd^ mice or pDC-ablated mice displayed higher or lower frequency of 5-bromo-2′deoxyuridine (BrdU)^+^ lymphocytes, including CD4^+^ T cells, CD8^+^ T cells and γδTCR^+^ T cells, as well as B220^+^ B cells in PLNs, than WT mice after the topical treatment of IMQ (Fig. 7b and Supplementary Fig. 14a). These results indicate that pDCs also control systemic inflammation and the proliferation of lymphocytes triggered by skin-mediated incorporation of IMQ.

IL-17-producing γδ T cells reportedly play a crucial role in the initiation of psoriasiform plaque formation^16^,^17^. IMQ-treated pDC-ablated mice exhibited a lower frequency of γδTCR^+^ T cells in skin-draining LNs than IMQ-treated WT mice and IMQ-treated *Siglech*^kd^ mice (Fig. 7c and Supplementary Fig. 14b). Furthermore, IMQ-treated *Siglech*^kd^ mice or IMQ-treated pDC-ablated mice showed a higher or lower proportion of IL-17-producing γδTCR^+^ T cells as well as IL-17-producing CD3^+^ T cells and T_H_17 cells in skin-draining LNs than IMQ-treated WT mice (Fig. 7c and Supplementary Fig. 14c,d). These results indicate that pDCs contribute to the generation of IL-17-producing γδTCR^+^ T cells and T_H_17 cells under TLR7-mediated inflammatory conditions. On the other hand, IMQ-treated *Ifnar1*^−/−^ mice displayed a reduced frequency of IFN-γ-producing T-cell subsets and an enhanced proportion of IL-17-producing T-cell subsets in skin-draining LNs compared with IMQ-treated WT mice (Supplementary Fig. 15a), indicating that the IFN-I signaling reciprocally controls the generation of IFN-γ-producing T-cell subsets and IL-17-producing T-cell subsets.

To clarify the role of the local activation of pDCs in skin damage for the initiation and exacerbation of psoriasis, we examined the expression of cytotoxic molecules in pDCs in skin-draining LNs of mice that had received the topical application of IMQ (Fig. 7d and Supplementary Fig. 14e,f). Interestingly, approximately 30% of B220^+^BST^+^ pDCs expressed Fas ligand (FasL) on the cell surface, whereas other leukocytes showed little or no expression of FasL in skin-draining LNs of IMQ-treated WT mice. We did not observe that any LN leukocytes expressed other cytotoxic molecules, such as TNF-related apoptosis-inducing ligand (TRAIL) and granzymes (data not shown). On the other hand, IMQ-treated *Siglech*^kd^ mice showed an increased frequency of FasL^+^ pDCs, whereas IMQ-treated pDC-ablated mice had a dramatic reduction of FasL^+^ leukocytes in skin-draining LNs compared with IMQ-treated WT mice. These results indicate that pDCs are involved in the induction of skin injury, possibly through the expression of FasL. We also observed that IMQ-treated *Ifnar1*^−/−^ mice not only showed a lower frequency of pDCs but also exhibited lower generation of FasL^+^ pDCs in skin-draining LNs than IMQ-treated WT mice (Supplementary Fig. 15b), indicating that IFN-I signaling is required for both the development of pDCs and their induction of the expression of FasL.

### pDCs aggravate pristane-induced lupus-like disease

**Figure 8:**
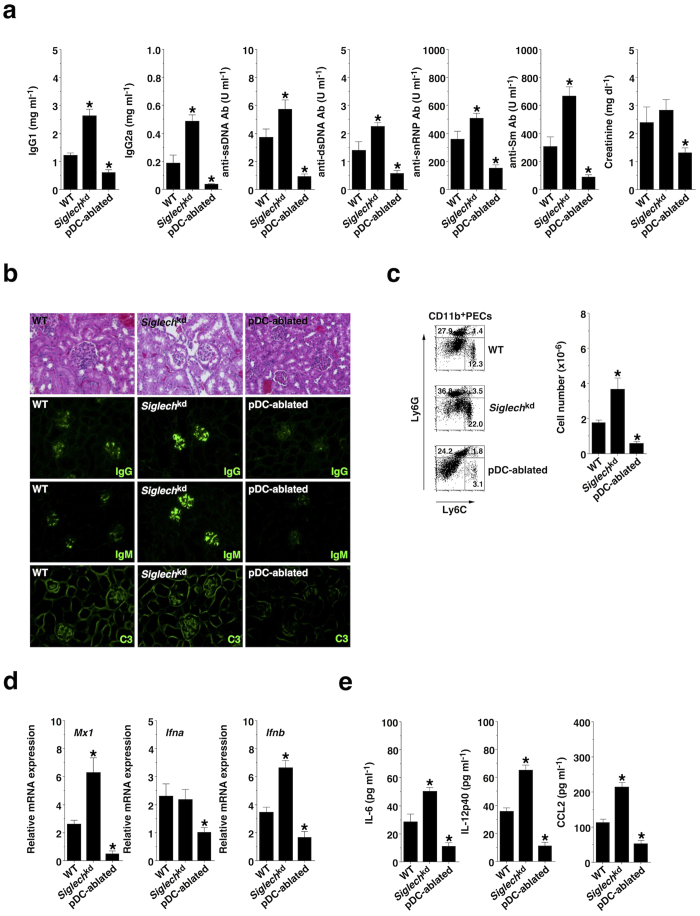
pDCs promote pristane-induced lupus-like disease. The BALB/c-background WT mice (n = 10), *Siglech*^kd^ mice (n = 10), and pDC-ablated mice (n = 10) were injected with × pristane. (**a**) Serum production of IgG1, IgG2a, anti-ssDNA Ab, anti-dsDNA Ab, anti-snRNP Ab, and anti-Sm Ab, and creatinine was measured 4 weeks after injection with pristane. (**b**) Glomerular immune complex and C3 deposits were determined 4 months after injection with pristane by H&E staining and immunofluorescence staining for IgG, IgM, and C3 (magnification: 40×). (**c–e**) PECs and peritoneal lavage fluid were collected 14 days after injection with pristane. (**c**) The frequency of Ly6C^+^ and/or Ly6G^+^ subsets among CD11b^+^ PECs (left panel) and their absolute number (right panel) were analyzed by flow cytometry. Data are presented as a dot plot, and numbers represent the proportion of positive cells in each quadrant (left panel). (**d**) Transcriptional expression of *Mx1*, *Ifna* and *Ifnb* in PECs was measured by quantitative RT-PCR. (**e**) Production of cytokines in peritoneal lavage fluid was measured by ELISA. Data are the mean ± s.d. from ten individual samples in a single experiment. *P < 0.01 compared with WT mice. All data are representative of at least three independent experiments.

Because excess IFN-I has been linked to the pathogenesis of SLE^19^,^20^,^21^, we explored the contribution of pDCs to the pathogenesis of pristane-induced lupus, in which both TLR7 and IFN signaling are required for the production of auto-Abs and the development of glomerulonephritis^40^,^41^,^42^. When compared with pristane-treated WT mice, pristane-treated *Siglech*^kd^ mice or pristane-treated pDC-ablated mice exhibited enhanced or reduced serum production of IgG1, IgG2a, anti-single-strand DNA (ssDNA) Ab, anti-double-strand DNA (dsDNA) Ab, anti-snRNP Ab, and anti-Sm Ab (Fig. 8a). Furthermore, pristane-treated pDC-ablated mice exhibited a lower serum level of creatinine, indicating renal dysfunction, than pristane-treated WT mice and pristane-treated *Siglech*^kd^ mice (Fig. 8a). While histopathologic assessment of the kidneys in pristane-treated WT mice showed enlarged hypercellular glomeruli, an increase in the mesangial matrix and mild peritubular mononuclear cell infiltrates, the development of the renal pathology was promoted or suppressed in pristane-treated *Siglech*^kd^ mice or pristane-treated pDC-ablated mice (Fig. 8b). Simultaneously, immunofluorescence analysis revealed that IgG, IgM and C3 deposition within the glomeruli was enhanced or reduced in pristane-treated *Siglech*^kd^ mice or pristane-treated pDC-ablated mice compared with those in pristane-treated WT mice (Fig. 8b). These results indicate that pDCs promote the development of pristane-induced lupus-like glomerulonephritis.

As Ly6C^high^ monocytes accumulated in the peritoneal cavity to produce IFN-I in this model of lupus^43^, we examined the correlation between pDCs and Ly6C^high^ monocytes. Interestingly, pristane-treated *Siglech*^kd^ mice or pristane-treated pDC-ablated mice displayed more or less accumulation of Ly6C^high^ monocytes in the peritoneal cavity than pristane-treated WT mice (Fig. 8c). Furthermore, Ly6C^high^ monocytes obtained from pristane-treated *Siglech*^kd^ mice or pristane-treated pDC-ablated mice showed higher or lower transcriptional expression of *Mx1*, *Ifna* and *Ifnb* as well as production of IL-6, IL-12p40 and CC chemokine ligand (CCL) 2 than those obtained from pristane-treated WT mice (Fig. 8d,e). These results indicate that pDCs control the peritoneal accumulation and activation status of Ly6C^high^ monocytes in the development of pristane-induced lupus-like disease.

## Discussion

While recent accumulating results suggest that pDCs are linked to the pathogenesis of psoriasis^24^ and SLE^25^,^26^,^27^, how pDCs control these IFN-I-associated autoimmune diseases remains unclear. In this study, we demonstrated a critical function for pDCs in the induction of TLR7-mediated innate and adaptive immune responses that cause autoimmune inflammation. In addition, our biochemical and genetic results clearly show that Siglec-H acts as an intrinsic “regulatory receptor” for the TLR7-mediated activation of pDCs that is important for regulation of the magnitude and quality of inflammation and T-cell responses.

Swiecki *et al.*^28^ have recently reported that Siglec-H-DTR-transgenic (Tg) mice, which were generated by bacterial artificial chromosome (BAC) recombineering, showed the conditional depletion of both pDCs and MZM expressing intracellular Siglec-H after DT injection. In line with our published results^13^, we confirmed that the administration with DT into *Siglech*^dtr/dtr^ KI mice led to the ablation of pDCs, but not MZM and other leukocytes, in lymphoid and peripheral tissues. The reason why the specificity of the ablated cell types differs between *Siglech*^dtr/dtr^ KI mice and Siglec-H-DTR-Tg mice might be the distinct expression pattern of DTR on the targeted cells, which could be influenced by the control of the endogenous versus transgenic promoter activity of the *Siglech* gene. Indeed, *Siglech*^dtr/dtr^ KI mice tolerated even repeated treatment with DT, whereas Siglec-H-DTR-Tg mice were susceptible to death induced by DT^28^, implying that essential nonhematopoietic cells also express DTR under control of the transgenic *Siglech* gene and generate fatal defects upon DT treatment. These discrepancies might explain some controversies regarding the influence of DT treatment on immune responses between *Siglech*^dtr/dtr^ KI mice and Siglec-H-DTR-Tg mice. On the other hand, *Siglech*^dtr/+^ KI mice showed the ablation of a fraction of CDP expressing Siglec-H^29^ as well as pDCs in BM upon DT injection. While some CDP expressed Siglec-H in BM, our results led us hypothesis that Siglec-H^−^ CDP might compensate the generation of cDCs because they retained the normal proportion of cDCs in lymphoid and peripheral tissues.

Analysis of *Siglech*^kd^ pDCs revealed that Siglec-H abrogated the TLR7-mediated production of IFN-I and IL-12p40, which was correlated with the impaired activation of the signaling cascades for IKK-α and NF-κB. Recent studies have reported the controversial effect of the deficiency of Siglec-H on the ability of pDCs to produce I-IFN in response to TLR9 ligand^12^,^28^, while the ligation of Siglec-H by an mAb on pDCs inhibited the TLR9-mediated production of I-IFN^44^. On the other hand, the inhibitory function of DAP-12 for TLR-mediated cytokine production in pDCs as well as other cell types reportedly required the immunoreceptor tyrosine-based activation motif (ITAM) within its cytoplasmic domain and presumably involved the recruitment and activation of the Syk tyrosine kinase, as a deficiency of Syk enhanced the response to TLR ligand^10^,^44^,^45^,^46^,^47^,^48^. Thus, Siglec-H could act as an intrinsic regulatory receptor to provide an inhibitory signal for TLR7-mediated downstream cascades to abrogate the cytokine production mediated through the activation of DAP12-Syk pathways. Taking these findings together, the engagement of Siglec-H by its unidentified endogenous ligand(s) could control the responsiveness of pDCs to endosomal TLR ligands. On the other hand, pDC-ablated mice manifested near-complete abrogation of IFN-I secretion and modest reduction of proinflammatory cytokine production following injection with the TLR7 ligand. Thus, pDCs could be the primary and major source of IFN-I, while they also might contribute to the proinflammatory cytokine release in response to TLR7 ligand *in vivo*. Collectively, these findings suggest that pDCs play a pivotal role in the initiation of endosomal TLR-mediated inflammation, and Siglec-H increases the threshold of responsiveness of pDCs to endosomal TLR ligands to prevent excessive harmful inflammation.

It has been suggested that pDCs are also involved in the induction of T-cell responses in the dependent of MHC-dependent presentation of Ag^49^,^50^ and the independently of direct Ag-presentation^51^ under certain inflammatory conditions *in vivo* while pDCs are known as poor APCs *in vitro* under steady-state conditions. We showed that pDCs enhanced the induction of T_eff_-cell responses when using antigenic protein plus TLR7 ligand for immunization, suggesting that the appropriate endosomal TLR ligands, but not other TLR ligands, are needed for licensing of pDCs in terms of the activation status for the induction of T_eff_-cell responses. On the other hand, the APC functions of cDCs were reduced in the absence of pDCs, whereas the deficiency of Siglec-H on pDCs promoted the maturational changes of cDCs under TLR7-mediated inflammatory conditions. Furthermore, the deficiency of IFNAR1 and CD40 suppressed the TLR7-mediated functional activation of cDCs and pDCs. Therefore, pDCs could provide help signals to cDCs for their optimal activation to initiate T_eff_-cell responses through the production of IFN-I as well as the interaction of CD40 and CD154^52^.

Whereas psoriasiform dermatitis reportedly occurs as a consequence of inflammation and T-cell responses^14^,^15^,^16^,^17^,^18^,^36^,^37^,^38^, the mechanism responsible for the initiation and development of the pathogenesis remains unclear. Furthermore, the role of pDCs in the development of psoriatic inflammation remains to be determined^14^,^15^,^24^,^53^,^54^,^55^. Analysis of gene-modified mice suggests that the initiation of IMQ-induced psoriasiform dermatitis involves the TLR7- and IFNAR1-mediated signaling cascades, whereas the activation of CD4^+^ T_eff_ cells through costimulation by APCs contributes to the disease progression. More importantly, both pDCs and cDCs are suggested to be required for the initiation and progression of psoriatic inflammation, in which the dermal infiltration of granulocytes and IL-17-producing γδTCR^+^ T cells as well as the activation of T cells and B cells under TLR7-mediated inflammatory conditions are dependent on Siglec-H-mediated control of pDC function. Interestingly, the TLR7-mediated expression of FasL on pDCs appeared to be associated with the apoptosis of neutrophils in pathogenic regions. These phenomena led us to hypothesize that pDCs act as primary cells to induce systemic and local inflammation upon the recognition of TLR7 ligands released from tissue cells under sterile pathophysiological conditions and/or microbes during infection. Subsequently, pDCs could activate cDCs and other leukocytes through the secretion of IFN-I as well as costimulation for the amplification of inflammation. Concomitantly, pDCs and cDCs cooperatively activate autoreactive naive T cells for the differentiation of T_eff_ cells to initiate psoriasiform dermatitis under TLR7-mediated inflammatory conditions. Furthermore, the activated pDCs could also infiltrate from skin-draining LNs into psoriatic skin to cause tissue damage, leading to the release of self DNA and antimicrobial peptides^15^ from host dying cells to form aggregated particles that trigger the further continuous activation of pDCs to produce the sustained level of IFN-I. On the other hand, the activated pDCs could downregulate the Siglec-H-mediated regulation due to its reduced expression^12^, which accelerates the psoriatic pathogenesis as potentiating pDC function under the deficiency of Siglec-H aggravates the disease severity. These scenarios might account for the establishment of chronic autoimmune skin inflammation. While the requirement of pDCs for the development of the psoriatic pathogenesis were confirmed by the reconstitution of pDC-ablated mice with pDCs, recent study suggested that pDCs are dispensable for the development of IMQ-induced skin inflammation^55^. This difference might be explained by experimental conditions, such as mouse strains, experimental period, environmental factors, and amount and interval of IMQ application.

In line with recent reports regarding the role of pDCs in SLE-prone animals^25^,^26^,^27^, our findings further suggests that the Siglec-H-mediated control of pDC function is crucial for the IFN-I-dependent progression of lupus-like disease as manifested by the impact on anti-self NA Ab production and glomerulonephritis. While the accumulated Ly6C^high^ monocytes have been previously suggested to be a major source of IFN-I production in the peritoneal cavity in an experimental lupus model^43^, pDCs appeared to control the recruitment of circulating Ly6C^high^ monocytes and their activation status under Siglec-H-mediated regulation. These observations suggest that pDCs not only function as upstream initiators of IFN-I-associated inflammation by activating Ly6C^high^ monocytes, but also trigger autoimmunity through unabated activation of cDCs depending on the IFNAR1- and CD40-mediated signaling, which stimulate autoreactive T cells and the differentiation of autoreactive B cells into Ab-secreting plasma cells for development of the pathogenesis of lupus-like disease.

In conclusion, we describe that pDCs play a crucial role in orchestrating the TLR7-mediated innate and adaptive immune responses under the intrinsic Siglec-H-mediated control for the initiation of IFN-I-associated autoimmune inflammation. Thus, pDCs represent an attractive potential therapeutic target for the amelioration of psoriasis and SLE.

## Methods

### Mice

The following mice at 8 to 12 weeks old were used in this study: C57BL/6 mice and BALB/c mice (Japan Clea), B6.CD45.1^+^OT-I TCR transgenic mice harboring OVA-specific CD8^+^ T cells^13^,^30^, B6.CD45.1^+^OT-II TCR transgenic mice harboring OVA-specific CD4^+^ T cells^13^,^30^, B6.*Ifnar1*^−/−^ mice^33^, B6.*Cd40*^−/−^ mice^34^, B6.*Tlr7*^−/−^ mice^31^, B6.FVB-Tg^*.Itgax-DTR/EGFP.57*^Lan/J (CD11c-DTR) mice (referred to as cDC-ablated mice)^39^, B6.*Cd80*^−/−^*Cd86*^−/−^ mice^35^ and B6.*Rag2*^−/−^γ*c*^−/−^ mice (The Jackson Laboratory). *Siglech*^dtr/dtr^ mice were generated as described previously^13^, and the mutant mice were cross-mated for more than twelve generations with C57BL/6 mice and BALB/c mice, and *Siglech*^+/+^ littermates were used as WT mice. In some experiments, B6.*Siglech*^dtr/+^ mice, in which pDCs and CDP retained the cell surface expression of Siglec-H, were also used to detect them. B6*.Siglech*^dtr/dtr^CD11c-DTR mice (referred to as cDC/pDCs-ablated mice) were bred in-house by crossing B6.*Siglech*^dtr/dtr^ mice with B6.CD11c-DTR mice. For systemic depletion of cDCs, *Cd11c*^dtr^-Tg mice were injected i.p. with DT (100 ng/mouse, Sigma-Aldrich) every 3–4 days. For the systemic ablation of pDCs, *Siglech*^dtr/dtr^ KI mice or *Siglech*^dtr/+^ KI mice were injected i.p. with DT (1 μg/mouse) every 3–4 day for 2 weeks (IMQ-induced psoriasiform skin inflammation) or 3 weeks (pristane-induced lupus). All mice were bred and maintained in specific pathogen-free conditions in the animal facility at University of Miyazaki, and all experiments were performed in accordance with institutional guidelines of the Animal Experiment Committee and Gene Recombination Experiment Committee.

### Cell isolation

To prepare single-cell suspensions from Spl and LNs, tissue samples were digested with collagenase type III (Worthington Biochemical) at 37 °C for 20 min, and were ground between glass slides. Splenocytes were treated with RBC lysis buffer (Sigma-Aldrich) before suspension. BM cells were flushed from the femurs and tibias. To prepare peritoneal exudate cells (PECs), the peritoneal cavity was lavaged with 10 ml of PBS using a 5-ml plastic syringe and 18-gauge needle. Single-cell suspensions were obtained by forcing through a 100-μm cell strainer (BD Biosciences). CD4^+^ T cells and CD8^+^ T cells were purified from Spl with mouse CD4 T lymphocyte Enrichment Set-DM and mouse CD8 T lymphocyte Enrichment Set-DM (both from BD Biosciences). CD11c^+^ DCs and BST2^+^ pDCs were purified by AutoMACS with mouse CD11c (N418) Microbeads and mouse anti-mPDCA-1 Microbeads (both from Miltenyi Biotec). Subsequently, CD11c^+^ DCs or BST2^+^ pDCs were sorted into CD11c^high^ cDCs or B220^+^BST2^+^ pDCs with high purity (each >99%) using a FACSAriaII cell sorter with fluorescein-conjugated mAbs (BD Biosciences). For preparation of MZM^56^, Spls were digested with a mixture of collagenase type III and DNase I (Sigma-Aldrich) at 37 °C for 25 min, then homogenized and filtered through a 100-μm cell strainer (BD Biosciences). Subsequently, splenocytes that had been depleted with T cells and B cells by CD90.2 (Thy1.2) Magnetic Particles-DM and CD45R/B220 Magnetic Particles-DM (both from BD Biosciences) were sorted into CD11b^+^F4/80^+^ macrophages using a FACSAriaII cell sorter with fluorescein-conjugated mAbs.

### Flow cytometry

Cells were stained with fluorescein-conjugated mAbs to mouse CD3α (145-2C11), CD4 (RM4–5), CD8α (53–6.7), CD11c (HL3), CD40 (3/23), CD44 (IM7), CD45.1 (A20), CD62L (MEL-14), CD80 (16-10A1), CD86 (GL1), CD154 (MR1), I-A/I-E (M5/114.15.2), B220 (RA3-6B2), Vα2 TCR (B20.1), γδTCR (GL3), IFN-γ (XMG1.2), IL-17A (TC11-18H10), isotype-matched control mAb (BD Biosciences), CD209b (22D1), CD253/TRAIL (N2B2) (eBioscience), CD11b (M1/70), CD178/FasL (MFL3), F4/80 (BM8), Ly6C (HK1.4), Ly6G (1A8), γδTCR (GL3) (Biolegend), mPDCA-1/BST2 (JF05-1C2.4.1) (Miltenyi Biotec), H-2K^b^ OVA tetramer (MBL) and Siglec-H (440c; Hycult Biotechnology). For the intracellular expression of cytokines, cells were incubated for 4 hrs with phorbol 12-myristate 13-acetate (PMA, 50 ng/ml; Sigma-Aldrich) plus ionomycin (500 ng/ml; Sigma-Aldrich) or OVA_257–264_ peptide (SIINFEKL; 10 μM) plus GolgiPlug (BD Biosciences) during the final 2 hrs. Subsequently, the cells were resuspended in Fixation-Permeabilization solution (BD Cytofix/Cytoperm kit; BD Biosciences) and intracellular cytokine staining was carried out according to the manufacturer’s directions. Fluorescence staining was analyzed with a FACSCalibur flow cytometer and CELLQuest software (both from BD Biosciences).

### Quantitative RT-PCR

Total RNA from pDCs (10^6^) was extracted with TRIzol (Life Technologies), and cDNA was synthesized with oligo (dT)_20_ as a primer using the PrimeScript™ RT Reagent Kit (Takara). Transcriptional expression levels were measured by real-time PCR (Thermal Cycler Dice Real Time System Single MRQ, Takara) using SYBR® *Premix Ex Taq*™ II (Tli RNaseH Plus) (Takara) with a pair of specific primers for *Siglech* (5′-aat tca cag aac tcc aca gc-3′ and 5′-gat ccc aag aag cag gaa tt-3′), *Ifna* (5′-tct gat gca gca ggt ggg-3′ and 5′-agg gct ctc cag act tct gct ctg-3′), *Infb* (5′-gca ctg ggt gga atg aga ct-3′ and 5′′-agt gga gag cag ttg agg aca-3′), *Mx1* (5′-gat ccg act tca ctt cca gat gg-3′ and 5′-cat ctc agt ggt agt caa ccc-3′) and *Gapdh* (5′-aaa ttc aac ggc aca gtc aag-3′ and 5′-tgg tgg tga aga cac cag tag-3′) after normalization for the expression of *Gapdh*.

### Culture of CD11c^+^ DCs

pDCs (10^5^) were cultured with or without IMQ (1.25–10 μg/ml; Invivogen) for 18 hrs in 48-well culture plates (BD Bioscience). The culture supernatants were collected and stored at -80^o^ C until assayed for cytokines.

### Detection of cytokines

Culture supernatants and sera were assayed for IFN-α, IFN-β (PBL), TNF-α, IL-6, IL-12p40, CCL2 (eBioscience), IL-17 A and IFN-γ (Biolegend) using ELISA kits according to the manufacturers’ instructions.

### Immunoblotting

pDCs (2×10^6^) were stimulated or not stimulated with IMQ (10 μg/ml) for the period indicated. Total lysate or the immunoprecipitate with anti-IKK-α Ab (2697; Santa Cruz Biotechnology) was analyzed by SDS-PAGE, and blots were probed with Ab for ERK (9102), JNK (9252), p38 (9212), IκBα (9242) (Cell Signaling Technology), IKK-α (M-280) and IRF-7 (H-246) (Santa Cruz Biotechnology) or the phosphospecific Ab for ERK (9101), JNK (9251), p38 (9211), IκBα (2859), IKK-α (2697) and IRF-7 (14767) (Cell Signaling Technology) followed by horseradish peroxidase (HRP)-conjugated goat anti-rabbit IgG (7074) (Cell Signaling Technology). The blot was visualized with an ECL Plus Western Blotting Detection System (GE Healthcare).

### *In vivo* TLR stimulation

Mice were intraperitoneally (i.p.) injected with IMQ (10 μg/mouse) with or without D-GalN (20 mg/mouse; Sigma-Aldrich), and sera were collected at the indicated times. In some experiments, Spl was obtained from the mice 24 hrs after injection.

### Ag presentation assay

CD45.1^+^ OT-I CD8^+^ T cells (10^5^) or CD45.1^+^ OT-II CD4^+^ T cells (10^5^) were cultured with the irradiated (15 Gy) cDCs or pDCs (10^4^) in the presence of OVA_257–264_ peptide (1 nM) or OVA_323-339_ peptide (ISQAVHAAHAEINEAGR; 1 μM) for 3 days in 96-well flat-bottomed plates. Alternatively, CD4^+^ T cells (2×10^5^) were cultured with irradiated (15 Gy) CD11c^+^ DCs (2×10^4^) in the presence or absence of OVA protein (1 mg/ml, Sigma-Aldrich) for 3 days in 96-well flat-bottomed plates (BD Biosciences). [^3^H]thymidine (GE Healthcare) incorporation was measured on day 3 for the last 18 hrs. In another experiment, the cells and the culture supernatants were collected to detect the production of cytokines.

### *In vitro* CD4^+^ T-cell differentiation assay

For the differentiation of T_H_1 cells^35^, CD45.1^+^OT-II CD4^+^ T cells (2×10^5^) were cultured with pDCs (2×10^4^) in the presence or absence of IMQ (5 μg/ml) in combination with OVA_323-339_ peptide (1 μM), anti-IL-4 mAb (10 μg/ml; 11B11, BD Biosciences) and recombinant mouse IL-2 (0.2 ng/ml; Wako Pure Chemicals) for 3 days in 96-well flat-bottomed plates. For the differentiation of T_H_17 cells^35^, CD45.1^+^OT-II CD4^+^ T cells (2×10^5^) were cultured with pDCs (2×10^4^) in the presence or absence of IMQ (5 μg/ml) in combination with OVA_323–339_ peptide (1 μM), anti-IFN-γ mAb (10 μg/ml; R4-6A2, BD Biosciences), anti-IL-4 mAb (10 μg/ml; 11B11), recombinant mouse IL-2 (0.2 ng/ml) and recombinant human transforming growth factor (TGF)-β1 (10 ng/ml; Wako Pure Chemicals) for 3 days in 96-well flat-bottomed plates. In some experiments, anti-IL-6 mAb (10 μg/ml; MP5-20F3, eBioscience), anti-IL-12 mAb (10 μg/ml; C17.8, eBioscience) or anti-IFNAR1 mAb (10 μg/ml; MAR1-5A3, eBioscience) was added to the culture. Analysis of the expression of IFN-γβ and/or IL-17 among gated CD4^+^ T cells was performed by flow cytometry as described above.

### Adoptive transfer

CD45.1^+^OT-I CD8^+^ T cells or CD45.1^+^OT-II CD4^+^ T cells were labeled with CFSE (Molecular Probes; 2.5 μM) at 37 °C for 10 min, and washed twice with cold PBS. Subsequently, CFSE-labeled CD45.1^+^OT-I CD8^+^ T cells or CD45.1^+^OT-II CD4^+^ T cells (5×10^6^/mouse) were i.v. injected into mice 24 hrs before the i.p. injection with or without OVA protein (50 μg/mouse) in combination with IMQ (10 μg/mouse). After 3 days, the gated CD45.1^+^OT-I CD8^+^ T cells and CD45.1^+^OT-II CD4^+^ T cells in Spl were analyzed for CFSE dilution to detect the dividing cells by flow cytometry. Alternatively, CD4^+^ T cells or *in vitro*-differentiated CD4^+^ T_eff_ cells (5×10^6^/mouse) prepared as described above were i.v. injected into mice *Rag2*^−/−^γ*c*^−/−^ mice.

### Immunization

For the analysis of Ag-specific CD4^+^ T-cell responses, mice were immunized subcutaneously (s.c.) with OVA protein (100 μg/mouse) plus IMQ (500 μg/mouse), and the Spl was obtained 14 days after the immunization. For the generation of antigen-specific CTLs^13^,^30^, mice received an i.p. injection of IMQ (10 μg/mouse) in combination with an i.p. injection of OVA protein (500 μg/mouse) plus anti-CD40 mAb (10 μg/mouse; 1C10; eBioscience), and Spl was obtained from the mice 6 days later.

### IMQ-induced psoriasis-like skin inflammation

A mouse model of IMQ-induced psoriasiform skin inflammation was established as previously^36^,^37^,^38^. In brief, mice were treated topically with either 62.5 mg of 5% IMQ cream (Mochida Pharmaceutical) or petrolatum (Wako Pure Chemicals) as a control on the shaved back and the right ear every other day for 14 days. The severity of inflammation of the back skin of each mouse was monitored daily as follows: erythema, scaling and thickening were scored independently on a scale from 0 to 4: 0, none; 1, slight; 2, moderate; 3, marked; and 4, very marked. The cumulative score (erythema plus scaling plus thickening) served as a measure of the severity of inflammation (scale 0–12).

### Pristane-induced lupus

BALB/c-background mice received a single i.p. injection of pristane (0.5 ml/mouse; 2,6,10,14-tetramethylpentadecane, Sigma-Aldrich)^40^. PECs and blood samples were obtained 2 and 4 weeks later, respectively. Kidneys were collected 4 months after treatment.

### Histopathologic assessment

Tissues were fixed with 4% paraformaldehyde (PFA) in PBS and embedded in paraffin. The sections of back skin and kidney (5 μm thickness) were stained with hematoxylin and eosin (H&E). The stained slides were examined with a BIOREVO fluorescence microscope (BZ-9000; KEYENCE). The areas of epidermis and dermis were quantified as thickness using ImageJ (National Institutes of Health) by a blinded observer as described previously^57^.

### Immunohistochemical analysis

For the detection of PCNA^58^, 4% PFA-fixed paraffin-embedded sections of back skin (5 μm thickness) were deparaffinized with toluene and rehydrated through a graded ethanol series. After inactivation of endogenous peroxidase with 0.3% H_2_O_2_ in methanol for 15 min and blocking with normal goat IgG, the sections were reacted overnight with the primary Ab against PCNA (PC10, Dako) in 1% BSA in PBS. After the reaction with HRP-conjugated goat anti-mouse IgG F(ab)’ second Ab, the sites of HRP were visualized with DAB and H_2_O_2_. For the detection of glomerular immune complex, kidney was embedded in OCT compound (Sakura Finetechnical) and frozen in liquid N_2_. The tissue segments were sectioned with a cryostat at 5 μm. Frozen sections were fixed in cold acetone and blocked with 5% normal rat serum. The direct immunofluorescence technique was performed using Alexa Fluor 488-conjugated goat anti-mouse IgG(H+L) and Alexa Fluor 488-conjugated goat anti-Mouse IgM (μ chain) (Life Technologies). Alternatively, the frozen sections were stained with rat anti-mouse C3 (Abcam) followed by Alexa Fluor 488-conjugated rabbit anti-rat IgG (Life Technologies). Sections incubated with an appropriate isotype control primary Ab served as controls. The stained slides were analyzed as described above.

### Hematological analysis

Sera were assayed for IgG1, IgG2a (eBioscience), anti-ssDNA Ab, anti-dsDNA Ab (Shibayagi), anti-snRNP Ab, and anti-Sm Ab (Alpha Diagnostic International), and creatinine (Cayman Chemical) according to the manufacturers’ instructions.

### *In vivo* BrdU labeling

For measurement of the proliferation of leukocytes *in vivo*, mice received i.p. injection of BrdU (1 mg; eBioscience), and the detection of the incorporated BrdU content in cells was performed 24 hrs after the injection flow cytometry using a BrdU staining kit for flow cytometry (eBioscience).

### Statistical analysis

Data are expressed as the mean ± s.d. from three to ten individual samples in a single experiment, and we perfomed at least three independent experiments. The statistical significance of the differences between the values obtained was evaluated by ANOVA and the Kaplan-Meier log-rank test. A P value of <0.01 was considered significant.

### Approval of experimental protocol

All experimental protocols were approved by institutional guidelines of the University of Miyazaki.

## Additional Information

**How to cite this article**: Takagi, H. *et al.* Plasmacytoid dendritic cells orchestrate TLR7-mediated innate and adaptive immunity for the initiation of autoimmune inflammation. *Sci. Rep.* **6**, 24477; doi: 10.1038/srep24477 (2016).

## Supplementary Material

Supplementary Information
