# Supplementary material for: Plasmacytoid dendritic cells orchestrate TLR7-mediated innate and adaptive immunity for the initiation of autoimmune inflammation
Source: Sci Rep. 2016 Apr 14;6:24477. doi: 10.1038/srep24477 (PMC4830934; doi:10.1038/srep24477)
Supplement: Supplementary Information [file srep24477-s1.pdf]

Supplementary Information

Plasmacytoid dendritic cells orchestrate TLR7-mediated innate and adaptive immunity for the initiation of autoimmune inflammation

Hideaki Takagi[1], Keiichi Arimura[1,2], Tomofumi Uto[1], Tomohiro. Fukaya[1], Takeshi Nakamura[1,3], Narantsog Choijookhuu[4], Yoshitaka Hishikawa[4] & Katsuaki Sato[1,5]

[1]Division of Immunology, Department of Infectious Diseases, Faculty of Medicine, University of Miyazaki, 5200 Kihara, Kiyotake, Miyazaki 889-1692, Japan

[2]Department of Oral and Maxillofacial Surgery, Faculty of Medicine, University of Miyazaki, 5200 Kihara, Kiyotake, Miyazaki 889-1692, Japan

[3]Department of Otolaryngology, Head and Neck Surgery, Faculty of Medicine, University of Miyazaki, 5200 Kihara, Kiyotake, Miyazaki 889-1692, Japan

[4]Division of Histochemistry and Cell Biology, Department of Anatomy, Faculty of Medicine, University of Miyazaki, Miyazaki 889-1692, Japan

[5]Japan Science and Technology Agency, Precursory Research for Embryonic Science and Technology (PRESTO), 4-1-8 Hon-cho, Kawaguchi, Saitama 332-0012, Japan

Correspondence should be addressed to K.S. (katsuaki_sato@med.miyazaki-u.ac.jp)

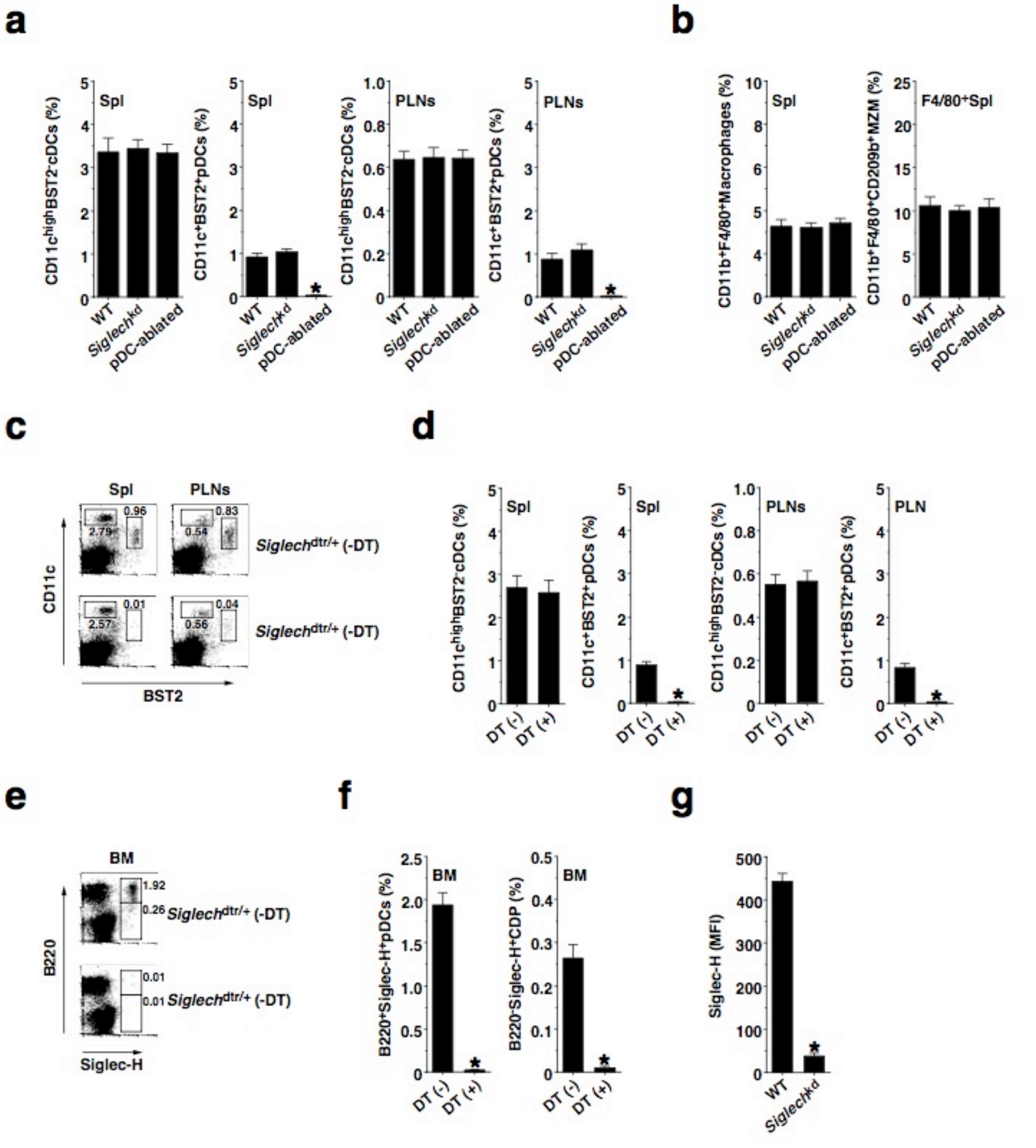

Supplementary Figure 1. Analysis of *Siglech*$^{dtr/dtr}$ mice and *Siglech*$^{dtr/+}$ mice. (**a**) The frequency of CD11c^{+}BST2^{+} pDCs in Spl and PLNs obtained from the C57BL/6-background WT mice (n=3), *Siglech*^{kd} mice (n=3), and pDC-ablated mice (DT-treated *Siglech*$^{dtr/dtr}$ KI mice, n=3) was analyzed by flow cytometry. Data are the mean percentage of positive cells ± s.d. from three individual samples in a single experiment. (**b**) The frequency of CD11b^{+}F4/80^{+}CD209b^{+} macrophages in Spl obtained from WT mice (n=3), *Siglech*^{kd} mice (n=3), and pDC-ablated mice (DT-treated *Siglech*$^{dtr/dtr}$ KI mice, n=3) was analyzed by flow cytometry. Data are the mean percentage of positive cells ± s.d. from three individual samples in a single experiment. (**c-f**) The frequency of CD11c^{high}BST2^{-} cDCs and CD11c^{+}BST2^{+} pDCs in Spl and PLNs (**c,d**) or B220^{+}Siglec-H^{+} pDCs and B220^{-}Siglec-H^{+} CDP in BM (**e,f**) obtained from the C57BL/6-background *Siglech*$^{dtr/+}$ KI mice that had been treated with or without DT

(each n=3) was analyzed by flow cytometry. (**c,e**) Data are presented as a dot plot, and numbers represent the proportion of $CD11c^{high}BST2^{-}$ cDCs (**c**), $CD11c^{+}BST2^{+}$ pDCs (**c**), $B220^{+}$Siglec-H^{+} pDCs (**e**), or $B220^{-}$Siglec-H^{+} CDP (**e**) among leukocytes in each quadrant. (**d,f**) Data are the mean percentage of positive cells ± s.d. from three individual samples in a single experiment. (**g**) Cell surface expression of Siglec-H on pDCs obtained from WT mice (n=3) and *Siglech*^{kd} mice (n=3) was measured by flow cytometry. Data are the mean MFI ± s.d. from three individual samples in a single experiment. All data are representative of at least three independent experiments.

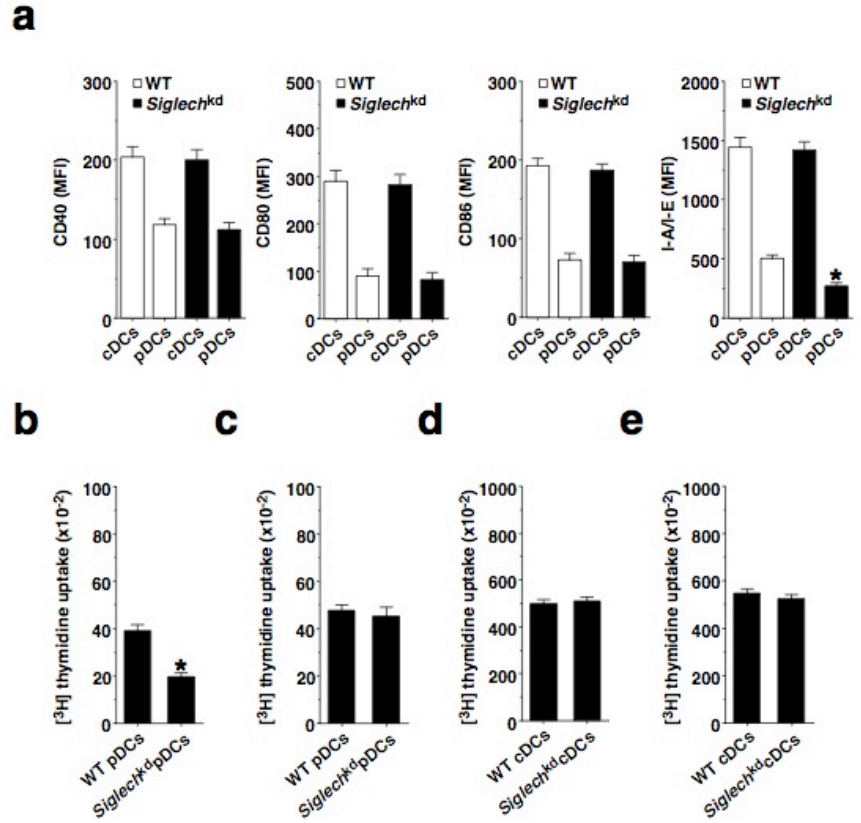

Supplementary Figure 2. Characterization of *Siglech*^{kd} pDCs under steady-state conditions. (**a**) The expression of MHC and costimulatory molecules on pDCs obtained from the C57BL/6-background WT mice (n=3) and *Siglech*^{kd} mice (n=3) was analyzed by flow cytometry. Data are MFI ± s.d. from three individual samples in a single experiment. (**b-e**) CD45.1 OT-II CD4$^+$ T cells (**b,d**) or CD45.1 OT-I CD8$^+$ T cells (**c,e**) were cultured with pDCs (**b,c**) or cDCs (**d,e**) obtained from the C57BL/6-background WT mice (n=3) and *Siglech*^{kd} mice (n=3) in the presence or absence of OVA$_{323-339}$ peptide (**b,d**) or OVA$_{257-264}$ peptide (**c,e**), and the proliferation was measured by [^{3}H]thymidine incorporation. Data are the mean ± s.d. from three individual samples in a single experiment. *P < 0.01 compared with WT mice. All data are representative of at least three independent experiments.

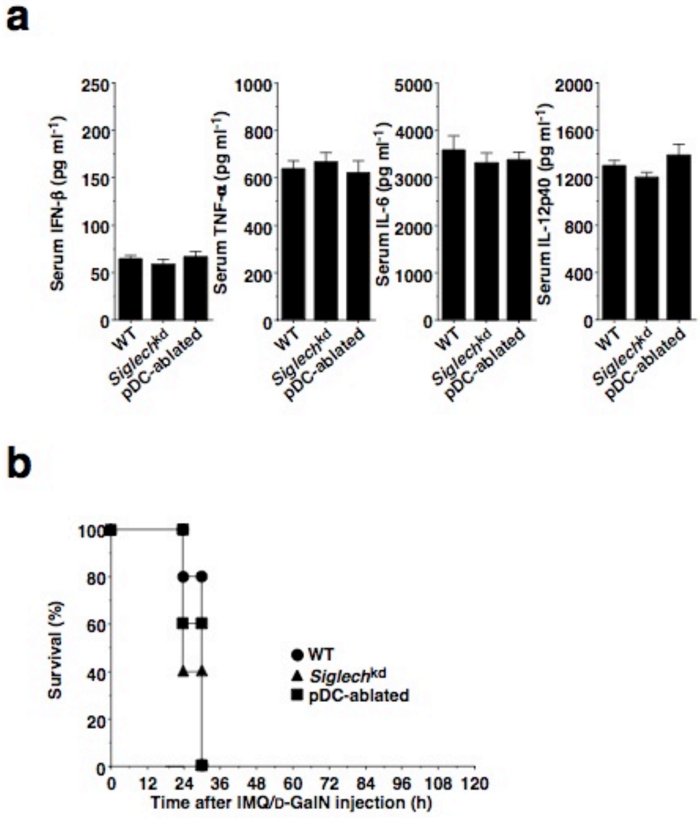

Figure 3. Role of pDCs in TLR4-mediated systemic lethal inflammation. The C57BL/6-background WT mice (n=10), *Siglech*^{kd} mice (n=10), and pDC-ablated mice (n=10) were injected with LPS plus D-GalN. (**a**) Serum production of cytokines was measured 24 hrs after injection with LPS plus D-GalN by ELISA. (**b**) Survival rate was monitored at the indicated times for 120 hrs after the injection of LPS plus D-GalN. Data are the mean ± s.d. from ten individual samples in a single experiment. *P < 0.01 compared with WT mice. All data are representative of at least three independent experiments.

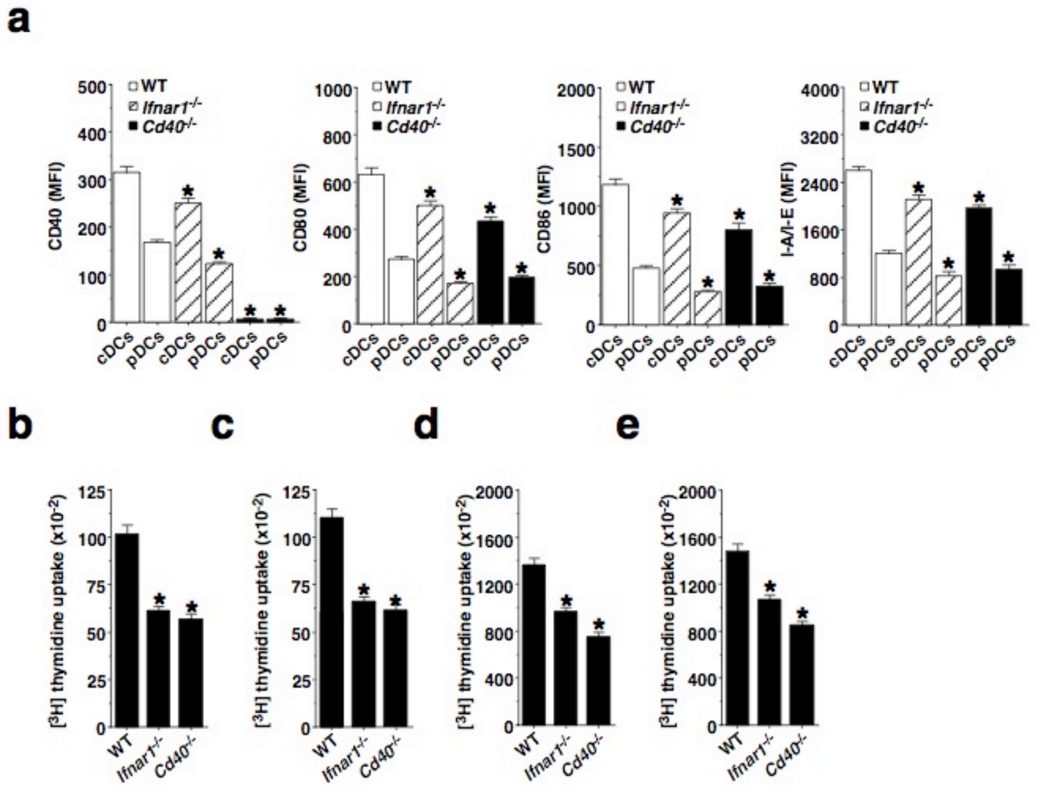

Supplementary Figure 4. IFN-I-IFNAR1 and CD40-CD154 regulate TLR7-mediated activation of pDCs and cDCs. The C57BL/6-background WT mice (n=6), the B6-background *Ifnar1*$^{-/-}$ mice (n=6), and the B6-background *Cd40*$^{-/-}$ mice (n=6) were injected with or without IMQ, and cDCs and pDCs were obtained 24 hrs after injection. (**a**) The expression of MHC and costimulatory molecules on cDCs and pDCs was analyzed by flow cytometry. Data are MFI ± s.d. from six individual samples in a single experiment. (**b-e**) CD45.1 OT-II CD4$^+$ T cells (**b**,**d**) or CD45.1 OT-I CD8$^+$ T cells (**c**,**e**) were cultured with pDCs (**b**,**c**) or cDCs (**d**,**e**) in the presence or absence of $OVA_{323-339}$ peptide (**b**,**d**) or $OVA_{257-264}$ peptide (**c**,**e**), and the proliferation was measured by [^{3}H]thymidine incorporation. Data are the mean ± s.d. from six individual samples in a single experiment. *P < 0.01 compared with WT mice. All data are representative of at least three independent experiments.

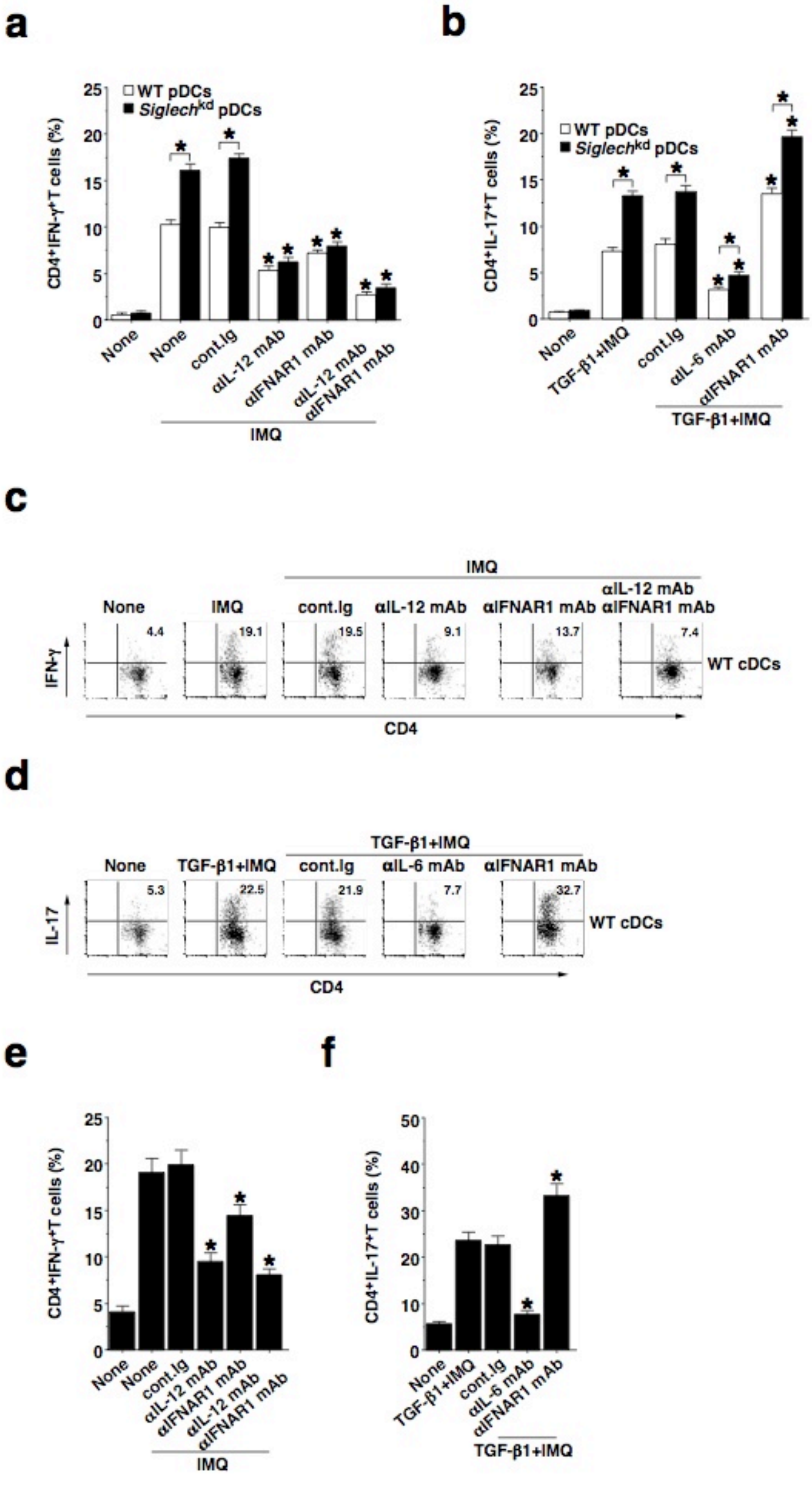

Figure 5. Siglec-H regulates the ability of pDCs to generate CD4$^+$ T$_{eff}$ cells under the TLR7-mediated inflammatory conditions. (**a**,**b**) CD45.1$^+$ OT-II CD4$^+$ T cells were cultured with pDCs obtained from the C57BL/6-background WT mice (n=3) and *Siglech*^{kd} mice (n=3) in the presence or absence of IMQ in combination with OVA$_{323-339}$ peptide and various blocking mAbs under T$_H$1 (**a**)- or T$_H$17 (**b**)-polarized culture

conditions for 3 days, and intracellular production of IFN-γ (**a**) or IL-17 (**b**) in the cultured $CD4^+$ T cells was analyzed by flow cytometry. Data are the mean percentage of positive cells ± s.d. from three individual samples in a single experiment. *P < 0.01 compared with IMQ (**a**), TGF-β1 plus IMQ (**b**), or among groups (**a,b**). (**c-f**) $CD45.1^+$ OT-II $CD4^+$ T cells were cultured with cDCs obtained from the C57BL/6-background WT mice (n=3) in the presence or absence of IMQ in combination with $OVA_{323-339}$ peptide and various blocking mAbs under T_H1 (**c,e**)- or T_H17 (**d,f**)-polarized culture conditions for 3 days, and intracellular production of IFN-γ (**c,e**) or IL-17 (**d,f**) in the cultured $CD4^+$ T cells was analyzed by flow cytometry. (**c,d**) Data are presented by a dot plot, and numbers represent the proportion of IFN-γ^+ cells (**c**) and IL-17^+ cells (**d**) among gated $CD4^+$ T cells in each quadrant. (**e,f**) Data are the mean percentage of positive cells ± s.d. from three individual samples in a single experiment. *P < 0.01 compared with IMQ (**e**) or TGF-β1 plus IMQ (**f**). All data are representative of at least three independent experiments.

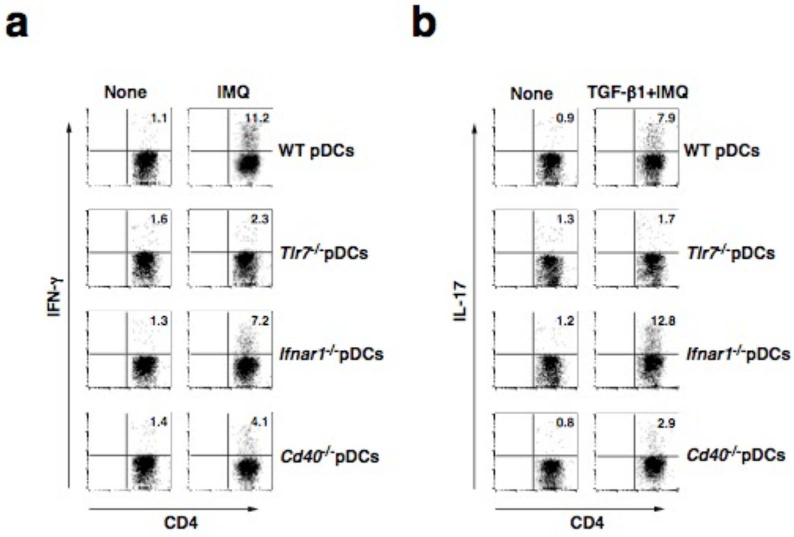

Supplementary Figure 6. IFN-I-IFNAR1 and CD40-CD154 regulate the ability of pDCs for TLR7-mediated generation of CD4$^+$ T$_{eff}$ cells. CD45.1$^+$ OT-II CD4$^+$ T cells were cultured with pDCs obtained from the C57BL/6-background WT mice, the B6-background *Tlr7*$^{-/-}$ mice, the B6-background *Ifnar1*$^{-/-}$ mice, and the B6-background *Cd40*$^{-/-}$ mice in the presence or absence of IMQ in combination with OVA$_{323\text{-}339}$ peptide under T$_H$1 (**a**)- or T$_H$17 (**b**)-polarized culture conditions for 3 days, and intracellular production of IFN-γ (**a**) or IL-17 (**b**) in the cultured CD4$^+$ T cells was analyzed by flow cytometry. Data are presented as a dot plot, and numbers represent the proportion of IFN-γ$^+$ cells (**a**) and IL-17$^+$ cells (**b**) among gated CD4$^+$ T cells in each quadrant. All data are representative of at least three independent experiments.

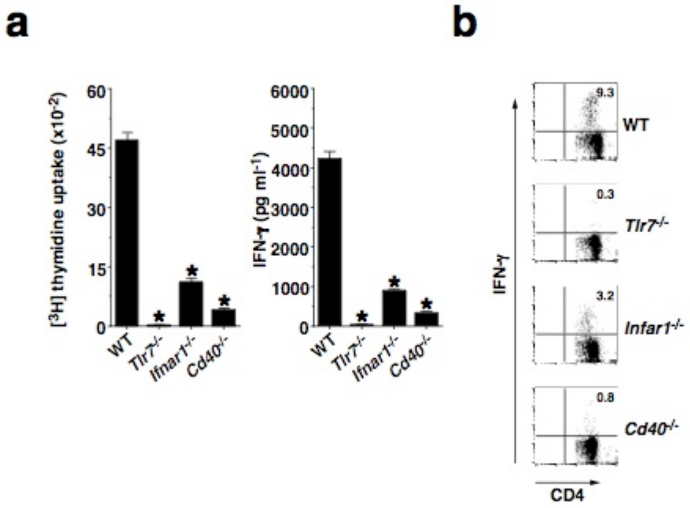

Supplementary Figure 7. IFN-I-IFNAR1 and CD40-CD154 regulate TLR7-mediated Ag-specific CD4$^+$ T-cell responses *in vivo*. The C57BL/6-background WT mice (n=6), the B6-background *Tlr7*$^{-/-}$ mice (n=6), the B6-background *Ifnar1*$^{-/-}$ mice (n=6), and the B6-background *Cd40*$^{-/-}$ mice (n=6) were immunized with OVA protein plus IMQ. At 14 days after the immunization, Spl CD4$^+$ T cells were cultured with WT CD11c$^+$ DCs in the presence or absence of OVA protein for the measurement of proliferative responses by [^{3}H]thymidine incorporation (**a**, left panel) and production of IFN-γ (**a**, right panel) by ELISA. (**b**) Intracellular production of IFN-γ in the cultured CD4$^+$ T cells was analyzed by flow cytometry. Data are presented as a dot plot, and numbers represent the proportion of IFN-γ$^+$ cells among gated CD4$^+$ T cells in each quadrant. Data are the mean ± s.d. from six individual samples in a single experiment. *P < 0.01 compared with WT mice. All data are representative of at least three independent experiments.

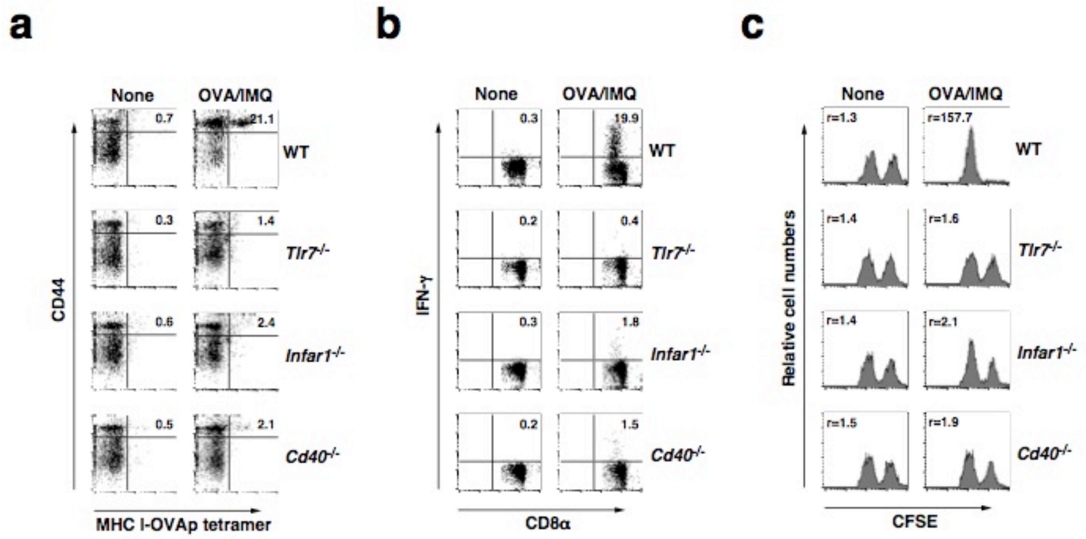

Supplementary Figure 8. IFN-I-IFNAR1 and CD40-CD154 regulate TLR7-mediated Ag-specific CD8$^+$ T-cell responses *in vivo*. the C57BL/6-background WT mice (n=6), the B6-background *Tlr7*$^{-/-}$ mice (n=6), the B6-background *Ifnar1*$^{-/-}$ mice (n=6), and the B6-background *Cd40*$^{-/-}$ mice (n=6) were immunized with OVA protein, IMQ and anti-CD40 mAb, and then a mixture of unpulsed CFSE^{low} cells plus Ag-pulsed CFSE^{high} cells was injected 5 days after the immunization. At 6 days after the immunization, splenocytes were analyzed for the generation of MHC I-OVA tetramer$^+$CD44^{high}CD8$^+$ T cells (**a**), for intracellular IFN-γ-producing CD8$^+$ T cells (**b**) and for cytotoxic activity *in vivo* (**c**) by flow cytometry. Data are presented as a dot plot (**a,b**), and numbers represent the proportion of MHC I-OVA tetramer$^+$CD44^{high} cells (**a**) and IFN-γ$^+$ cells (**b**) among gated CD8$^+$ T cells in each quadrant, or by a histogram (**c**), and numbers represent the ratio of unpulsed CFSE^{low} cells to Ag-pulsed CFSE^{high} cells in each histogram. All data are representative of at least three independent experiments.

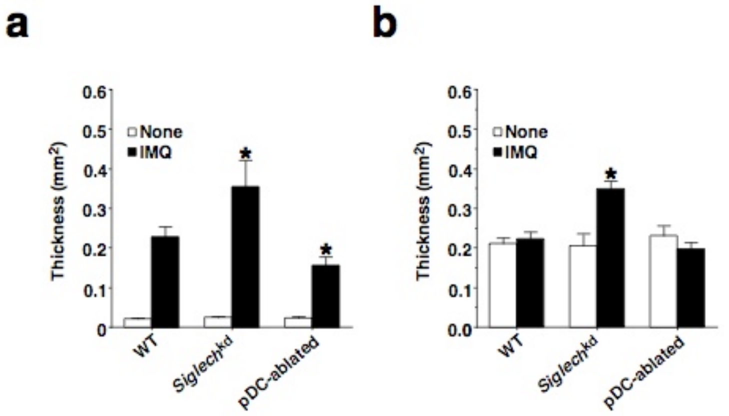

Supplementary Figure 9. pDCs promotes IMQ-induced psoriasiform dermatitis. H&E staining of the paraffin-embedded sections obtained from the back skin of untreated mice and mice on day 6 days after treatment with IMQ cream as prepared in Fig. 6e, and the areas of epidermis (**a**) and dermis (**b**) were quantified as thickness. Data are the mean ± s.d. from ten individual samples in a single experiment. *P < 0.01 compared with WT mice. All data are representative of at least three independent experiments.

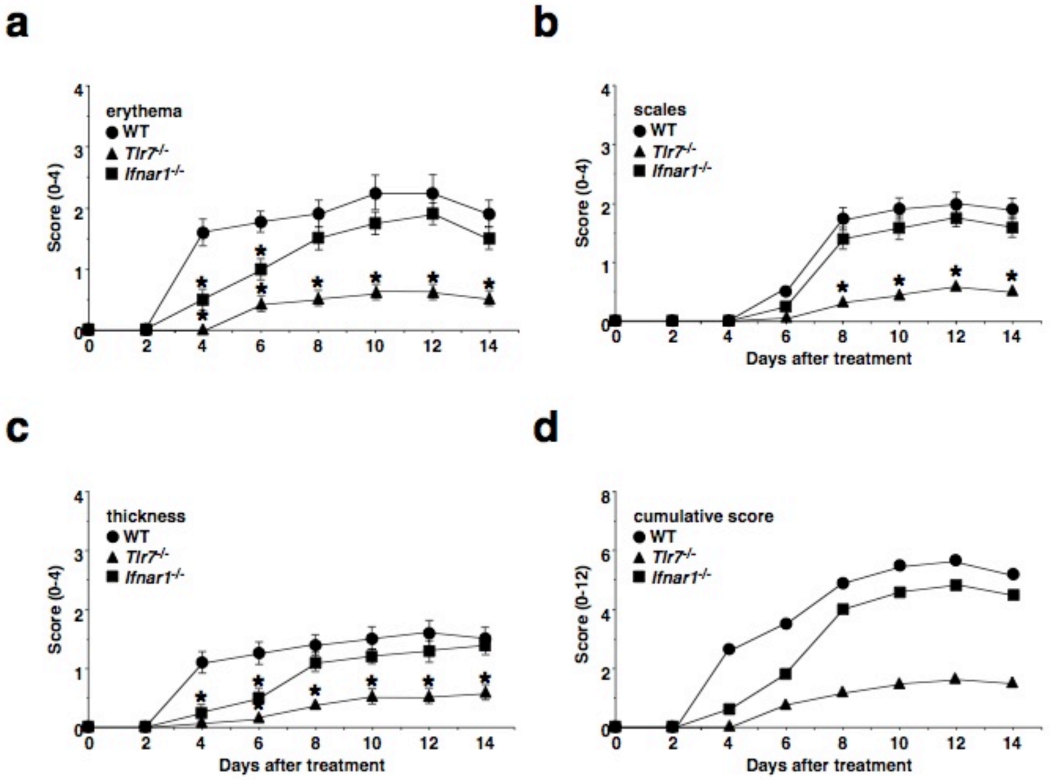

Supplementary Figure 10. Roles of TLR7 and IFN-I-IFNAR1 in the development of IMQ-induced psoriasiform dermatitis. The C57BL/6-background WT mice (n=10), the B6-background *Tlr7*$^{-/-}$ mice (n=10), and the B6-background *Ifnar1*$^{-/-}$ mice (n=10) were treated topically with IMQ cream on the shaved back every other day for 14 days. The disease severity of each mouse was scored daily, and erythema (**a**), scaling (**b**), thickness (**c**) and cumulative score (**d**) of the back skin at the indicated times were plotted. Data are the mean ± s.d. from ten individual samples in a single experiment. *P < 0.01 compared with WT mice. All data are representative of at least three independent experiments.

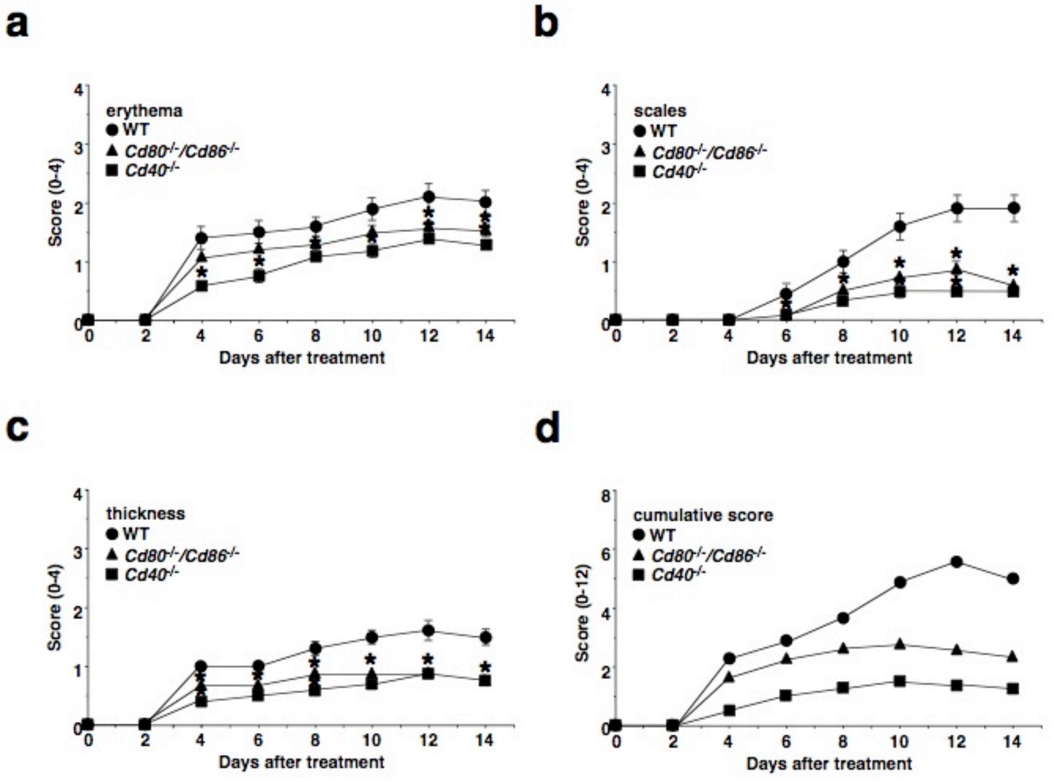

Supplementary Figure 11. Roles of CD80/CD86 and CD40 in IMQ-induced psoriasiform dermatitis. The C57BL/6-background WT mice (n=10), the B6-background *Cd80*$^{-/-}$*Cd86*$^{-/-}$ mice (n=10), and the B6-background *Cd40*$^{-/-}$ mice (n=10) were treated topically with IMQ cream on the shaved back every other day for 14 days. The disease severity of each mouse was scored daily, and erythema (**a**), scaling (**b**), thickness (**c**) and cumulative score (**d**) of the back skin at the indicated times were plotted. Data are the mean ± s.d. from ten individual samples in a single experiment. *P < 0.01 compared with WT mice. All data are representative of at least three independent experiments.

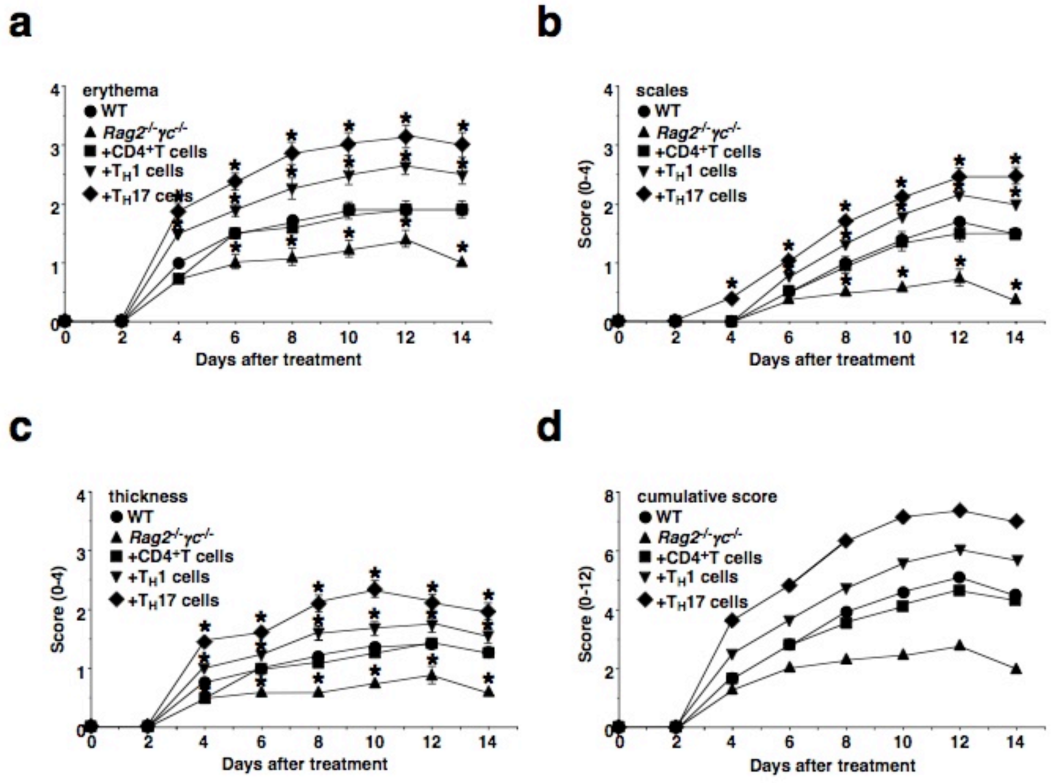

Supplementary Figure 12. Roles of T_H1 cells and T_H17 cells in IMQ-induced psoriasiform dermatitis. The C57BL/6-background WT mice (n=10), The B6-background *Rag2*$^{-/-}$*γc*$^{-/-}$ mice (n=10), and the B6-background *Rag2*$^{-/-}$*γc*$^{-/-}$ mice that had been inoculated with $CD4^+$ T cells (n=10), T_H1 cells (n=10) and T_H17 cells (n=10) were treated topically with IMQ cream on the shaved back every other day for 14 days. The disease severity of each mouse was scored daily, and erythema (**a**), scaling (**b**), thickness (**c**) and cumulative score (**d**) of the back skin at the indicated times were plotted. Data are the mean ± s.d. from ten individual samples in a single experiment. *P < 0.01 compared with WT mice. All data are representative of at least three independent experiments.

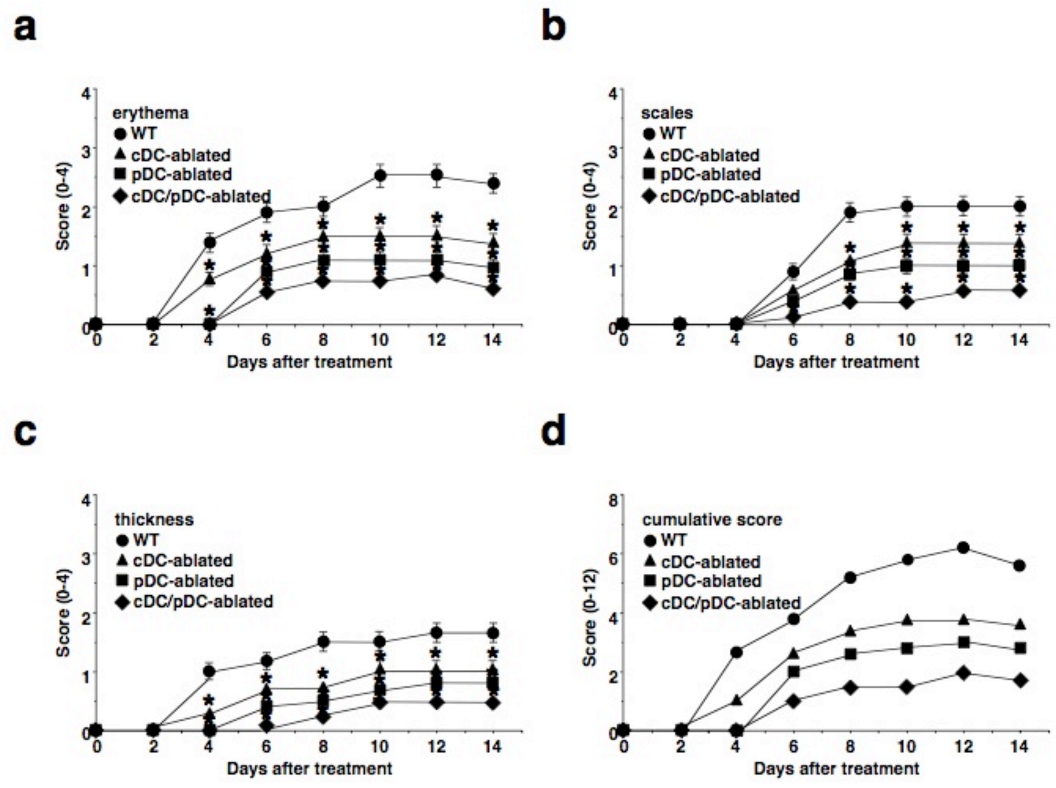

Supplementary Figure 13. Role of cDCs in IMQ-induced psoriasiform dermatitis. The C57BL/6-background WT mice (n=10), cDC-ablated mice (n=10), pDC-ablated mice (n=10), and cDC/pDC-ablated mice (n=10) were treated topically with IMQ cream on the shaved back every other day for 14 days. The disease severity of each mouse was scored daily, and erythema (**a**), scaling (**b**), thickness (**c**) and cumulative score (**d**) of the back skin at the indicated times were plotted. Data are the mean ± s.d. from ten individual samples in a single experiment. *P < 0.01 compared with WT mice. All data are representative of at least three independent experiments.

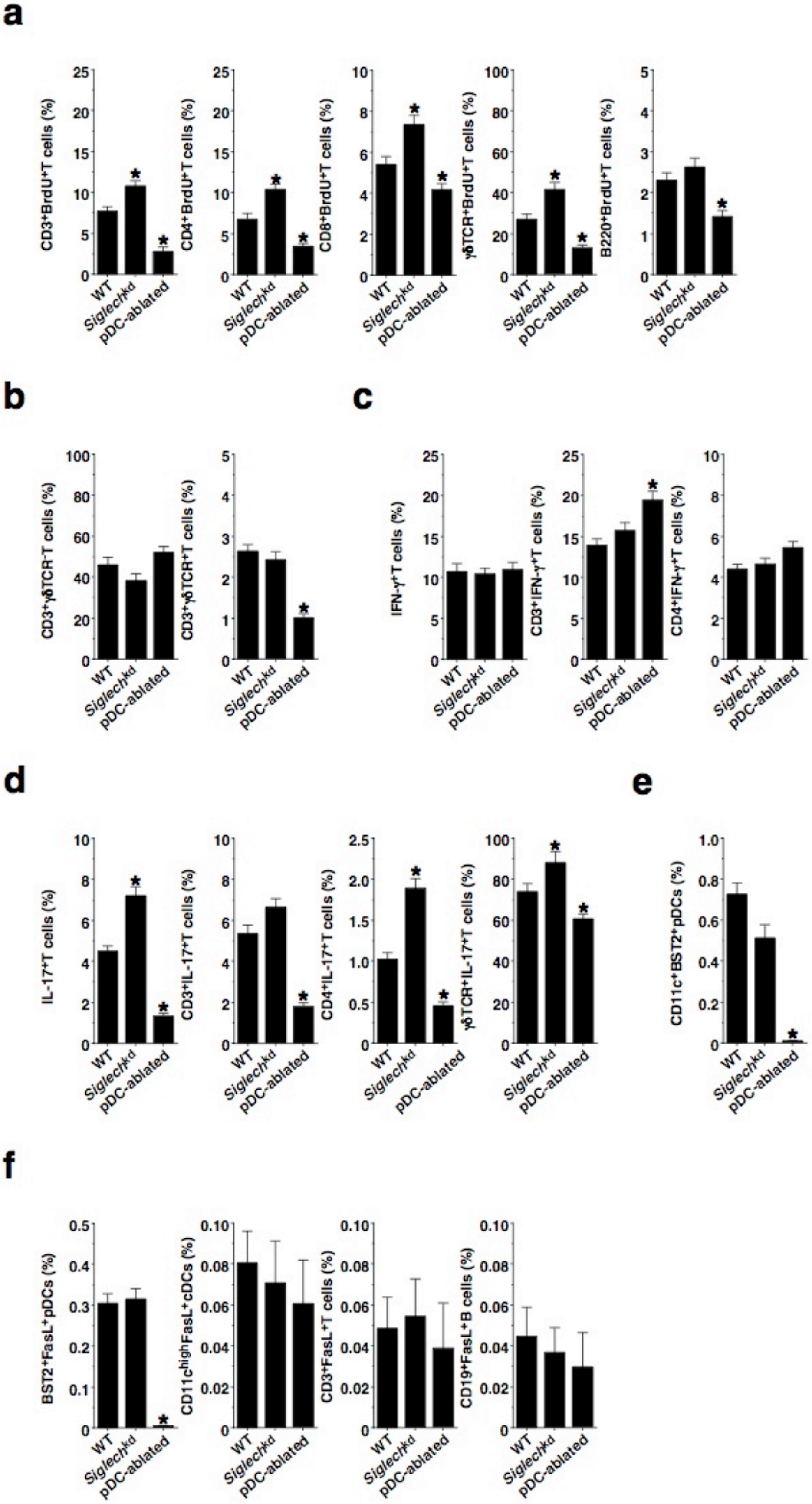
a
CD3+BrdU+T cells (%)
CD4+BrdU+T cells (%)
CD8+BrdU+T cells (%)
γδTCR+BrdU+T cells (%)
B220+BrdU+T cells (%)
b
CD3+γδTCR-T cells (%)
CD3+γδTCR+T cells (%)
c
IFN-γ+T cells (%)
CD3+IFN-γ+T cells (%)
CD4+IFN-γ+T cells (%)
γδTCR+IFN-γ+T cells (%)
d
IL-17+T cells (%)
CD3+IL-17+T cells (%)
CD4+IL-17+T cells (%)
γδTCR+IL-17+T cells (%)
e
CD11c+BST2+pDCs (%)
f
BST2+FasL+pDCs (%)
CD11chighFasL+cDCs (%)
CD3+FasL+T cells (%)
CD19+FasL+B cells (%)
WT
Siglechkd
pDC-ablated
*

Supplementary Figure 14. pDCs control IMQ-induced T-cell responses in skin-draining LNs. The C57BL/6-background WT mice (n=6), *Siglech*^{kd} mice (n=6), and pDC-ablated mice (n=6) were treated topically with IMQ cream on the shaved back every other day for 6 days. (**a**) The frequency of $BrdU^+$ lymphocyes in skin-draining LNs was analyzed 6 days after treatment with IMQ cream by flow cytometry. Data are the mean percentage of positive cells ± s.d. from six individual samples in a single experiment. (**b-d**) The constituency of T-cell subsets in skin-draining LNs (**b**) and their intracellular production of IFN-γ (**c**) and IL-17 (**d**) were analyzed 6 days after treatment with IMQ cream by flow cytometry. Data are the mean percentage of positive cells ± s.d. from six individual samples in a single experiment. (**e,f**) The proportion of $CD11c^+BST2^+$ pDCs (**e**) and $FasL^+$ leukocytes (**f**) in skin-draining LNs was analyzed 6 days after treatment with IMQ cream by flow cytometry. Data are the mean percentage of positive cells ± s.d. from six individual samples in a single experiment. *P < 0.01 compared with WT mice. All data are representative of at least three independent experiments.

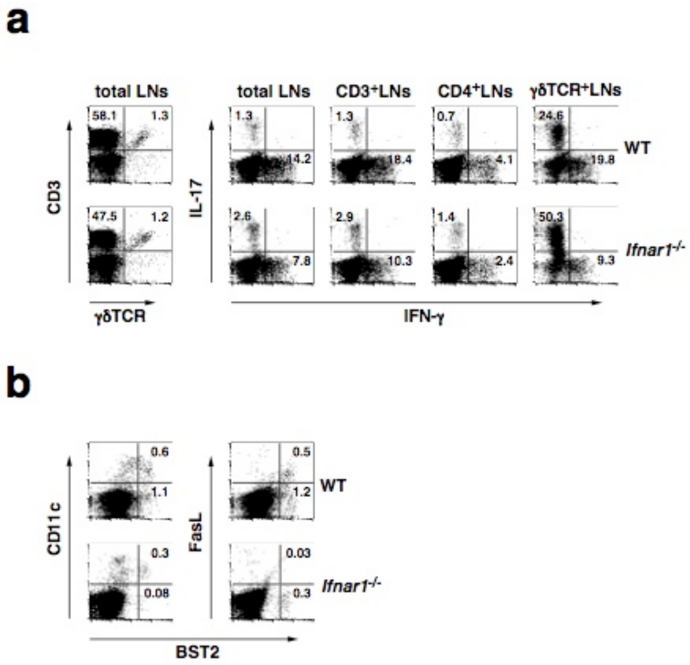

Supplementary Figure 15. Roles of IFN-I-IFNAR1 in IMQ-induced local inflammatory responses. The C57BL/6-background WT mice (n=6) and the B6-background *Ifnar1*$^{-/-}$ mice (n=6) were treated topically with IMQ cream on the shaved back every other day for 6 days. (**a**) The constituency of T-cell subsets in skin-draining LNs (left panel) and their intracellular production of IFN-γ and IL-17 (right panel) were analyzed by flow cytometry. Data are presented as a dot plot, and numbers represent the proportion of positive cells in each quadrant. (**b**) The expression of FasL and cell surface molecules on leukocytes in skin-draining LNs was analyzed by flow cytometry. Data are presented as a dot plot, and numbers represent the proportion of positive cells in each quadrant. All data are representative of at least three independent experiments.
